# Atomically Fe‐anchored MOF‐on‐MOF nanozyme with differential signal amplification for ultrasensitive cathodic electrochemiluminescence immunoassay

**DOI:** 10.1002/EXP.20220151

**Published:** 2023-07-07

**Authors:** Chuanping Li, Tianxiang Hang, Yongdong Jin

**Affiliations:** ^1^ State Key Laboratory of Electroanalytical Chemistry Changchun Institute of Applied Chemistry Chinese Academy of Sciences Changchun People's Republic of China; ^2^ Anhui Laboratory of Functional Coordinated Complexes for Materials Chemistry and Application Anhui Polytechnic University Wuhu People's Republic of China; ^3^ School of Applied Chemistry and Engineering University of Science and Technology of China Hefei People's Republic of China

**Keywords:** electrochemiluminescence, immunoassay, MOF‐on‐MOF, nanozyme

## Abstract

The successful application of electrochemiluminescence (ECL) in immunoassays for clinical diagnosis requires stable electrodes and high‐efficient ECL signal amplification strategies. Herein, the authors discovered a new class of atomically dispersed peroxidase‐like nanozymes with multiple active sites (CoNi‐MOF@PCN‐224/Fe), which significantly improved the catalytic performance and uncovered the underlying mechanism. Experimental studies and theoretical calculation results revealed that the nanozyme introduced a Fenton‐like reaction into the catalytic system and the crucial synergistic effects of definite active moieties endow CoNi‐MOF@PCN‐224/Fe strong electron‐withdrawing effect and low thermodynamic activation energy toward H_2_O_2_. Benefiting from the high peroxidase‐like activity of the hybrid system, the resultant ECL electrode exhibited superior catalytic activity in the luminol‐H_2_O_2_ system and resulted in an ≈17‐fold increase in the ECL intensity. In addition, plasmonic Ag/Au core‐satellite nanocubes (Ag/AuNCs) were designed as high‐efficient co‐reactant quenchers to improve the performance of the ECL immunoassay. On the basis of the differential signal amplification strategy (DSAS) proposed, the immunoassay displayed superior detection ability, with a low limit of detection (LOD) of 0.13 pg mL^−1^ for prostate‐specific antigen (PSA). The designed atomically anchored MOF‐on‐MOF nanozyme and DSAS strategy provides more possibilities for the ultrasensitive detection of disease markers in clinical diagnosis.

## Graphical abstract

An ultrasensitive cathodic electrochemiluminescence immunoassay was developed by designing atomically Fe‐anchored MOF‐on‐MOF nanozymes (CoNi‐MOF@PCN‐224/Fe) as ECL electrode materials and plasmonic Ag/AuNCs as high‐efficient co‐reactant quenchers. The proposed differential signal amplification strategy endowed the immunoassay with superior detection ability, which provide more possibilities for the ultrasensitive detection of disease markers in clinical diagnosis.

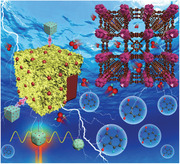

## 1 INTRODUCTION

Electrochemiluminescence (ECL), inheriting the advantages of chemiluminescence and electrochemistry, is regarded as a promising analytical technique owing to its low background, wide dynamic range, and high sensitivity.^[^ ^1^, ^2^, ^3^ ^]^ Since the first report of the ECL phenomenon by Bard et al.,^[^ ^4^ ^]^ numerous ECL luminophores, such as metal‐organic frameworks (MOFs),^[^ ^5^, ^6^ ^]^ luminol,^[^ ^7^, ^8^ ^]^ Ru(bpy)_3_^2+[^ ^9^, ^10^ ^]^ and nanomaterials^[^ ^11^, ^12^ ^]^ have been extensively investigated. Among them, luminol has attracted considerable interest on account of its acceptable price, low oxidation potential and high quantum yield.^[^ ^13^ ^]^ So far studies of luminol‐based ECL applications are mostly focused on its anodic emission. Nevertheless, anodic ECL is commonly suffered from numerous interference reactions due to the coexisting reductive components.^[^ ^14^ ^]^ Theoretically, cathodic ECL possesses simpler emission mechanisms and more negative excitation potential, which is beneficial for ECL detection applications. However, luminol can hardly be activated at a negative potential, which led to relatively weak cathodic ECL signals and resulted in limited applications.^[^ ^15^ ^]^

In the classical luminol system, H_2_O_2_ is commonly used as a co‐reactant to generate reactive oxygen species (e.g., •O_2_^−^ and •OH) to promote luminol oxidation and enhance ECL intensity.^[^ ^15^ ^]^ In this process, the co‐reactant introduced plays a key role in generating reactive intermediate radicals from co‐reactants and promoting the ECL intensity of luminophores. Recently, Nanozymes with peroxidase‐mimic activity have been discovered to be able to catalyze the H_2_O_2_ and produce •O_2_^−^ and •OH with high efficiency.^[^ ^16^, ^17^, ^18^ ^]^ Thereinto, metal‐organic framework (MOF)‐based nanozymes constructed with inorganic metal ions (cluster) and organic linkers are promising candidates with excellent characteristics of a well‐defined coordination network, tunable porosity and high surface area.^[^ ^19^ ^]^ To date, only a few MOF nanozymes exhibited peroxidase activity, enabling cascade reactions with high catalytic activity. Nevertheless, the reported MOF nanozymes still suffer from poor catalytic activity due to weak electron transfer kinetics underlying the heterogeneous reactions of the co‐reactant and limited accessible active sites of individual MOF.^[^ ^20^, ^21^ ^]^ MOF‐on‐MOF nanozymes, which integrate multiple anisotropic MOFs into one unit, are ideal alternatives to increase the active sites and promote catalytic activity on account of their heterogeneous components and diverse structures.^[^ ^22^ ^]^ However, few attempts have been made to design and apply MOF‐on‐MOF nanozymes for ECL applications.

To achieve ultrasensitive biodetection, nanoengineering of ECL nanoprobe and the development of signal‐enhanced detection strategies are highly desired. At present, resonance energy transfer (ECL‐RET) is the most commonly used tactic to quench ECL signal and achieve the detection of analytes.^[^ ^23^, ^24^ ^]^ However, in the ECL‐RET scheme, it is quite difficult to achieve a complete overlap between the emission spectrum of the donor and the absorption spectrum of the acceptor. The spectral mismatch seriously affects the energy transfer and ECL signal amplification. Besides, the intermolecular ECL‐RET may result in massive energy loss in the energy transfer process, which limits the signal amplification intensity and detection sensitivity.

**SCHEME 1:**
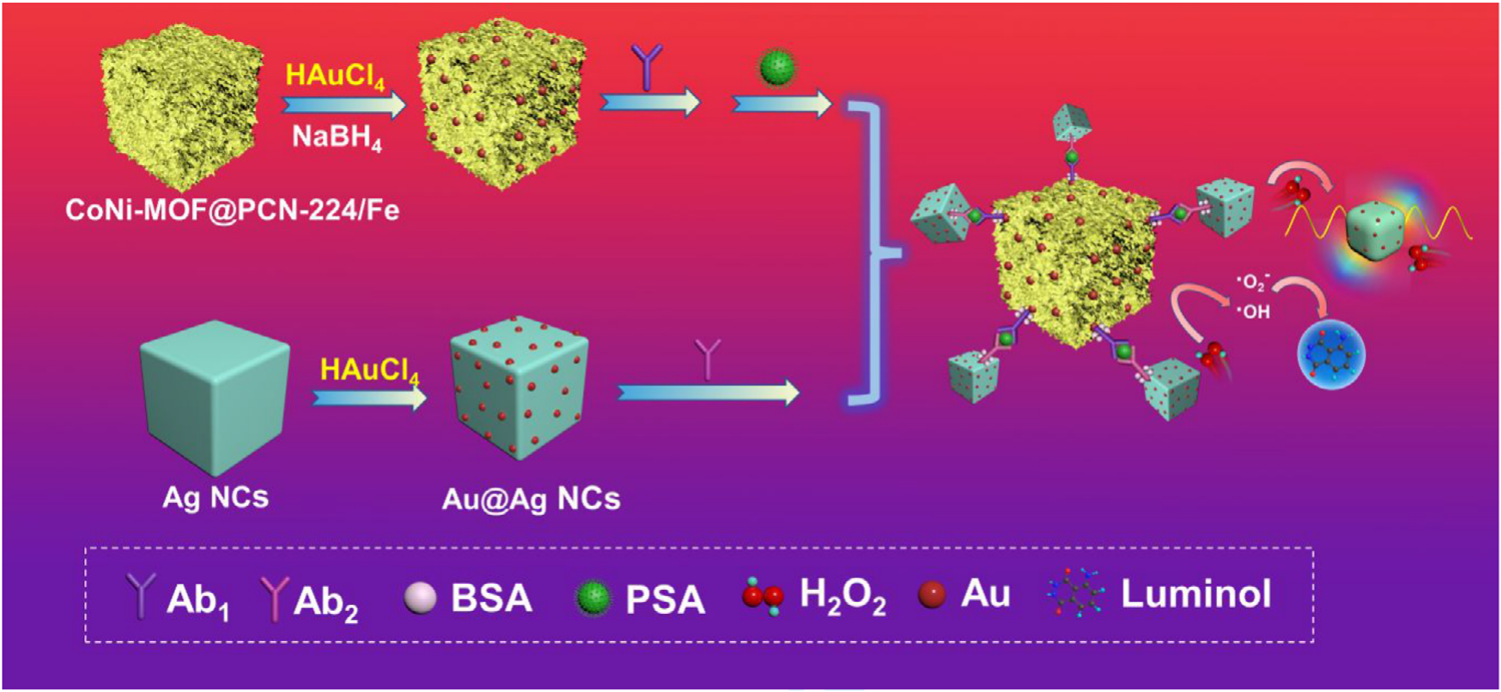
Schematic diagram of the preparation of CoNi‐MOF@PCN‐224/Fe MOF‐on‐MOF nanozyme and proposed differential signal amplification strategy for PSA detection.

Considering the technical bottlenecks mentioned above, herein, we developed a highly‐efficient cathodic ECL detection platform by synchronously introducing MOF‐on‐MOF nanozymes and a differential signal amplification strategy (DSAS). Firstly, an MOF‐on‐MOF nanozyme (CoNi‐MOF@PCN‐224/Fe) was designed and nano‐engineered to introduce a Fenton‐like reaction via an atom‐anchoring strategy. The designed CoNi‐MOF@PCN‐224/Fe exhibited excellent peroxidase‐like activity in the luminol‐H_2_O_2_ system due to the multiple active sites (both PCN‐224/Fe and CoNi‐MOF) and low activation energy barriers confirmed by DFT calculations. Besides, the Ag/Au core‐satellite nanocubes (Ag/AuNCs) as efficient co‐reactant (H_2_O_2_) quenchers were also designed and prepared. In the presence of antigen (e.g., PSA), the co‐reactant (H_2_O_2_) will be consumed by etching the antigen‐labeled plasmonic Ag/AuNCs and thus decrease the ECL intensity (Scheme 1). The synergy of ECL intensity enhancement derived from the Fe atom‐anchored MOF‐on‐MOF nanozyme catalysis and efficient ECL signal quenching via co‐reactant consumption (called differential signal amplification strategy, DSAS) is proved to be an efficient strategy to achieve ultrasensitive biodetection, by using prostate‐specific antigen (PSA) as a specific model. This work provides a new signal amplification strategy for ECL bioassay and will promote practical clinical applications of MOFs in ultrasensitive immunoassays.

## 2 RESULTS AND DISCUSSION

### 2.1 Theoretical calculations of the activity of the Fenton‐like nanozyme

**FIGURE 1:**
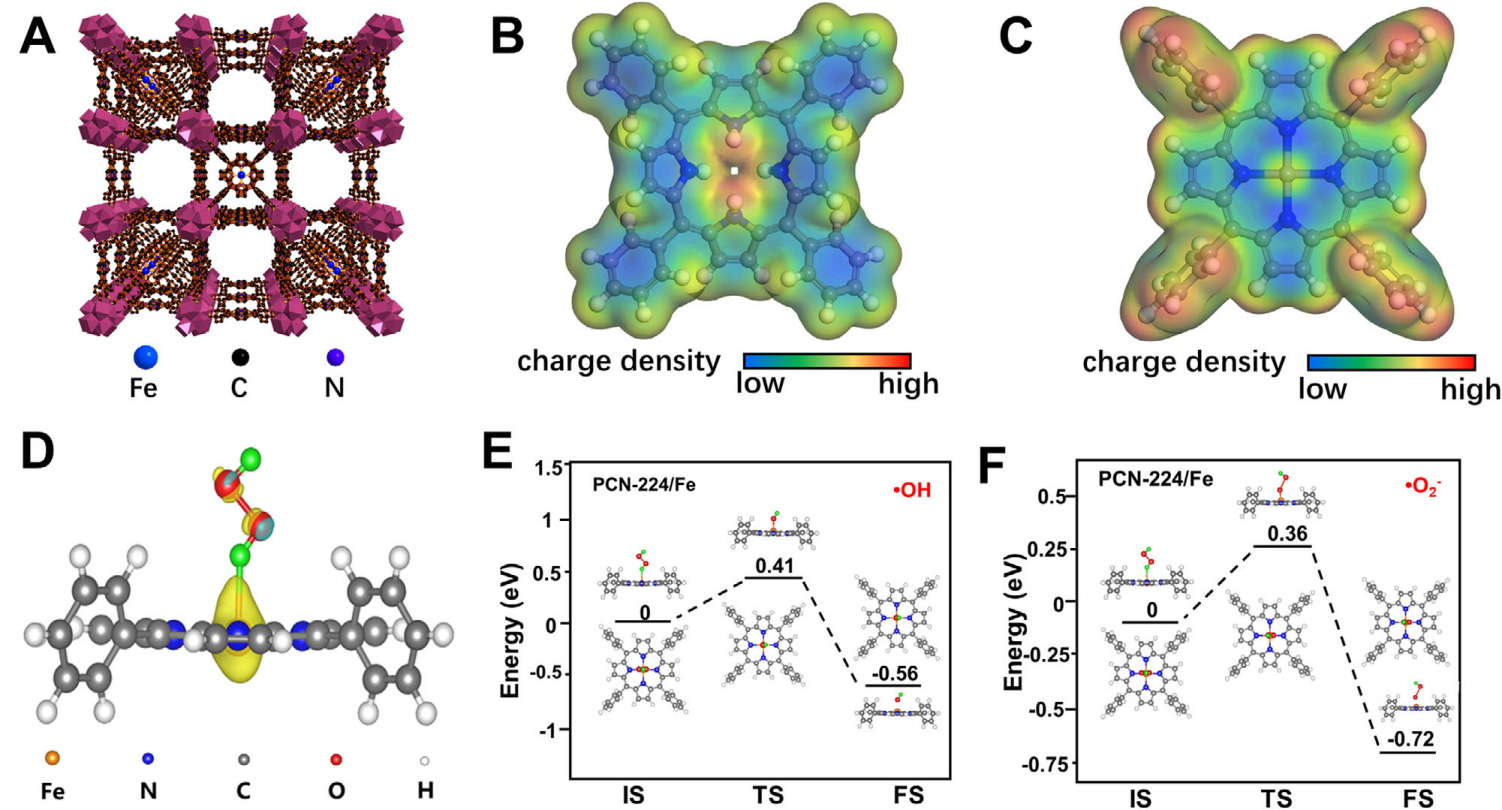
DFT calculations. (A) Structure of the PCN‐224/Fe, charge‐density diagrams of (B) PCN‐224 and (C) PCN‐224/Fe, (D) differential charge density distribution between PCN‐224/Fe and H_2_O_2_, reaction mechanism diagram and free energy scheme of the generation of (E) •OH and (F) •O_2_^−^ on PCN‐224/Fe.

**FIGURE 2:**
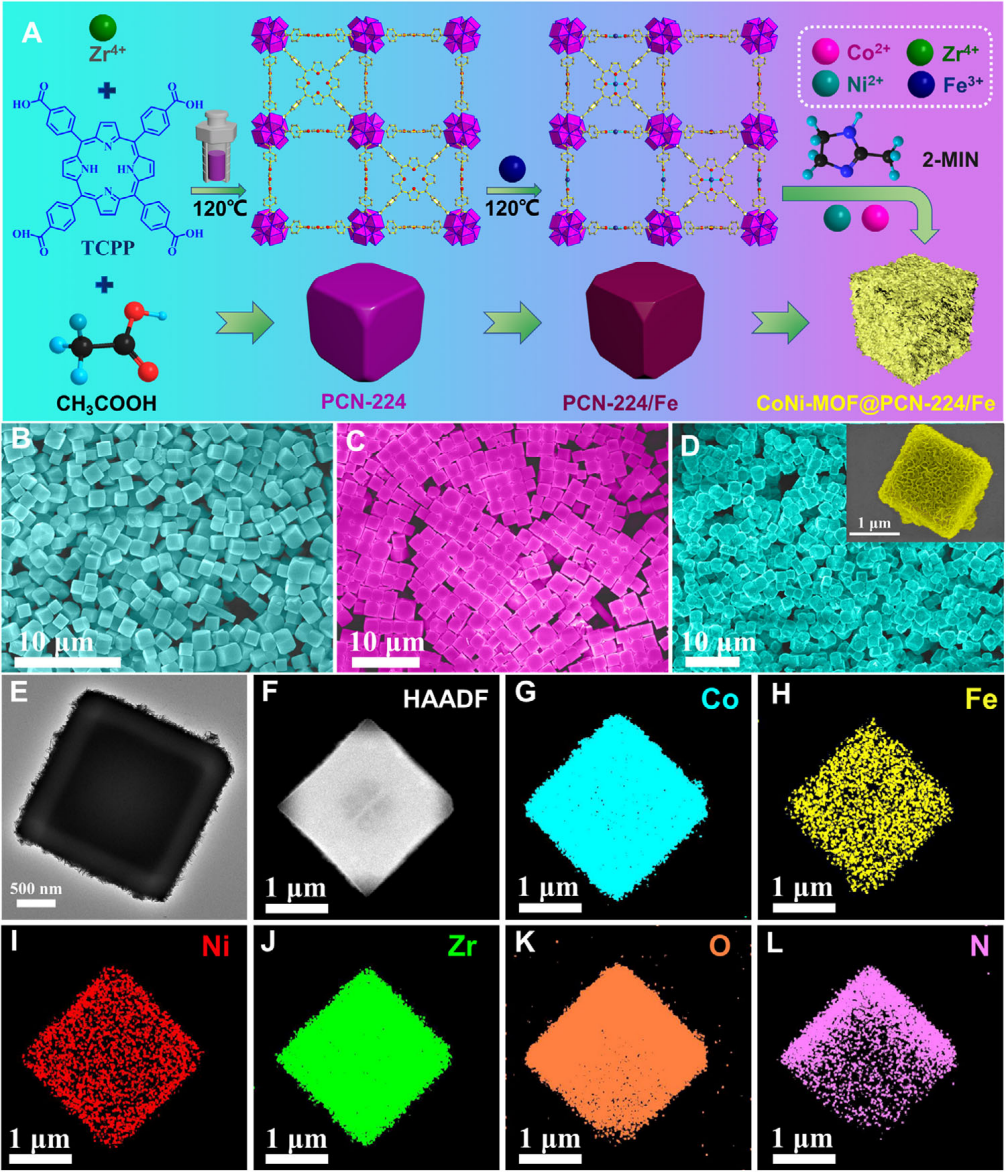
Morphology characterizations. (A) Schematic diagram of the synthetic procedures of CoNi‐MOF@PCN‐224/Fe; SEM images of (B) PCN‐224, (C) PCN‐224/Fe and (D) CoNi‐MOF@PCN‐224/Fe. Inset: Enlarged SEM image of CoNi‐MOF@PCN‐224/Fe. (E) TEM, (F) HAADF‐STEM and (G–L) elemental mapping of CoNi‐MOF@PCN‐224/Fe.

Recently, the Fenton reaction has been widely exploited for applications in many fields, such as photodynamic therapy of cancer, pollutant degradation, etc.^[^ ^25^, ^26^ ^]^ In the reaction, with the participation of iron ions, H_2_O_2_ can be efficiently decomposed and converted to reactive oxygen species (ROS, e.g., •OH and •O_2_^−^). Thus, introducing the Fenton reaction into porous MOFs to construct highly‐efficient nanozymes with multiple active sites is expected to be an ideal strategy to achieve a high ECL signal in the luminol‐H_2_O_2_ system. Inspired by the metalloporphyrin of natural hemin, PCN‐224, which is constructed by Zr_6_ cluster and Tetrakis(4‐carboxyphenyl)porphyrin (TCPP), would be an ideal MOF structure as the Fe^3+^ can reside in the center of porphyrin ligand via FeN_4_ to afford hemin‐like nanozyme activity, as shown in Figure 1A. The atomically Fe‐anchored PCN‐224 (PCN‐224/Fe) is expected to provide an optimized electronic state and behave with superior activity to generate various ROS to further enhance the ECL signal. To confirm this conjecture, density functional theory (DFT) calculation was first conducted. Figure 1B,C is the charge–density diagram, which describes the spatial charge distribution of the nanozymes. After Fe^3+^ anchoring, the electron density distribution changes greatly, in which an electron‐deficient center is obviously formed around Fe^3+^. Moreover, the PCN‐224/Fe shows a strong electron‐withdrawing effect (the yellow electron clouds represent electron enrichment) toward H_2_O_2_, as shown in the differential charge density distribution (Figure 1D). Such structure and electron transfer are expected to be in favor of regulating the activation energy for the reaction intermediates and therefore changing the catalytic reaction rate. Figure 1E,F, Figure S1 and Equation (S1) are the free energy schemes and chemical mechanism during the H_2_O_2_ decomposition. The activation energy barriers of H_2_O_2_ decomposition to produce •OH and •O_2_^−^ are calculated to be 0.41 eV and 0.36 eV on PCN‐224/Fe, which is much lower than that on pristine PCN‐224 (0.87 eV for •OH and 0.54 for •O_2_^−^). The result indicates that the dissociation of H_2_O_2_ is more thermodynamically favorable on PCN‐224/Fe than PCN‐224 and the Fenton‐like reaction can accelerate the catalytic reaction theoretically and further promote the production of •OH and •O_2_^−^ as expected by the design. However, nanozymes with single‐metal active sites usually suffered from limited catalytic activity due to the restricted accessible active sites.^[^ ^19^, ^27^ ^]^ Recently, multi‐metal‐site catalysts were found to exhibit superior catalytic activity compared with single‐metal ones, and this property is assumed to be the result of the synergetic effect of the coexistence of multi‐metal sites on optimizing the interaction between active sites and reactants.^[^ ^28^, ^29^ ^]^ In recent year, Cobalt‐ or nickel‐based metal‐organic frameworks were found to have peroxidase‐like property. By precisely controlling the composition, structure and morphology, the Cobalt‐ or nickel‐based MOFs could exhibit high‐efficient catalytic activities in biosensing, cancer treatment, environmental protection etc.^[^ ^27^, ^30^ ^]^ Inspired by this, we tried to further introduce highly active CoNi‐MOF nanosheet into the PCN‐224/Fe systems and constructed MOF‐on‐MOF nanozymes with multi‐metal sites (CoNi‐MOF@PCN‐224/Fe, Scheme 1 and Figure 2A). The as‐prepared nanozymes are expected to integrate the merits of each active site and will obtain high‐performance ECL sensing applications in immunoassay.

### 2.2 Morphology and structure characterizations of the nanozymes

Figure 2A shows the chemical preparation procedures of the CoNi‐MOF@PCN‐224/Fe. First, PCN‐224 was synthesized by using a facile hydrothermal method. As displayed in Figure 2B, the PCN‐224 exhibits a uniform cubic morphology. The rich carboxylic and conjugate benzene groups of PCN‐224 were revealed by Fourier Transform Infrared (FTIR) analysis in Figure S2.^[^ ^31^ ^]^ Moreover, the powder X‐ray diffraction (PXRD) pattern of the nanocubes indicated the successful preparation of PCN‐224 (Figure S3). Then the Fe^3+^ was implanted into PCN‐224 via a solvothermal process. With the insertion of Fe^3+^, the morphology and structure remain intact without any visible changes (Figure 2C). Subsequently, CoNi‐MOF was in‐situ covered on the surface of PCN‐224/Fe, showing an ultrathin nanosheet structure (Figure 2D). These dense ultrathin nanosheets further increased the exposed active sites and hence the catalytic activity of the nanozymes. As seen from scanning electron microscope (SEM) and transmission electron microscopy (TEM) images in Figure 2D,E, the typical size of the as‐prepared CoNi‐MOF@PCN‐224/Fe is ≈2 μm. Energy dispersive spectrum (EDS) analysis (Figure S4), HAADF‐STEM (Figure 2F) and elemental mapping images (Figure 2G–I) indicate that the elements of Co, Fe, Ni, Zr, O and N are uniformly distributed, which evidenced the successful preparation of the MOF‐on‐MOF structures of CoNi‐MOF@PCN‐224/Fe.

**FIGURE 3:**
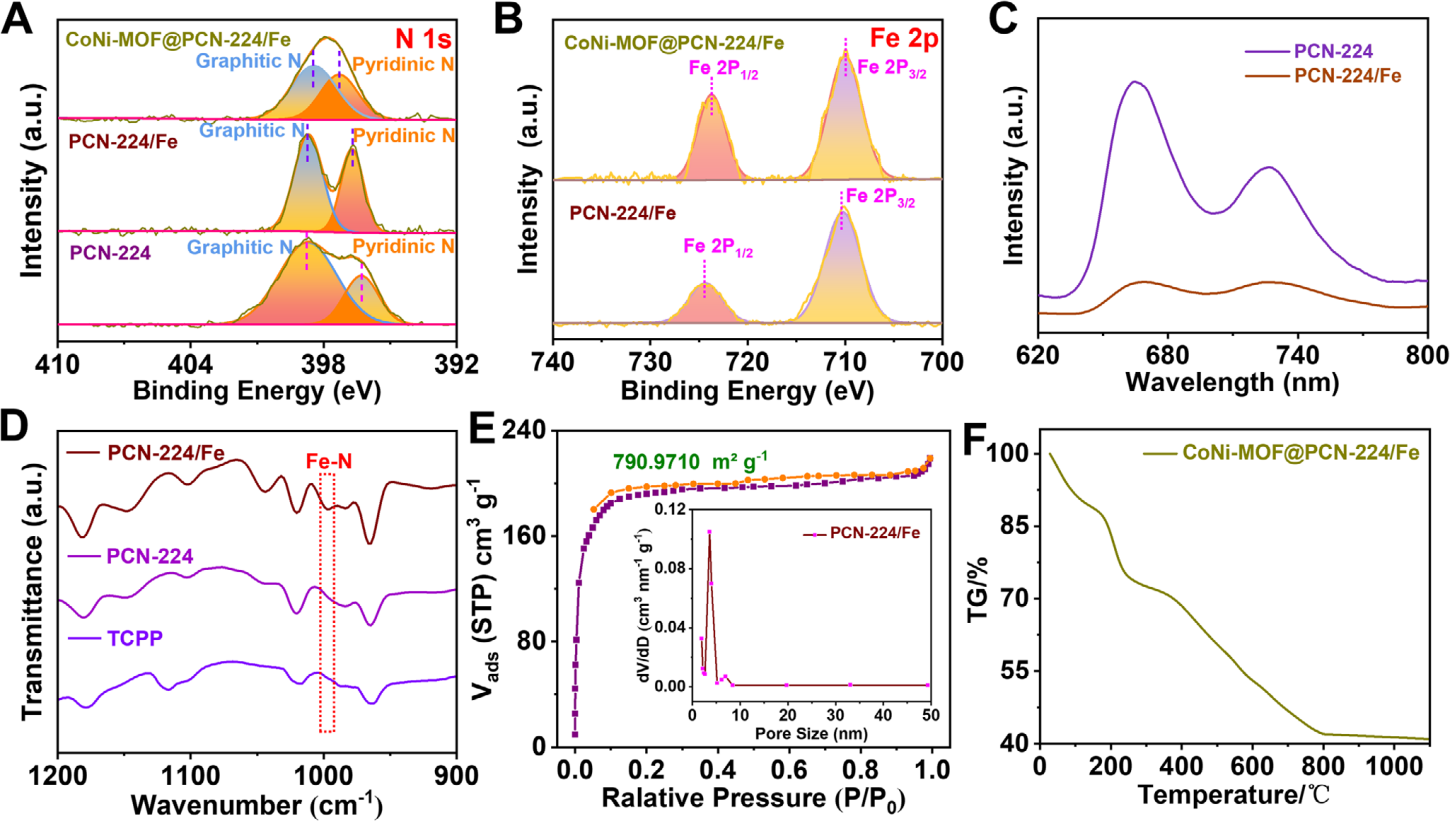
Characterizations of CoNi‐MOF@PCN‐224/Fe. XPS of (A) N 1s, and (B) Fe 2p and (C) the fluorescence spectra of PCN‐224 and PCN‐224/Fe; (D) Fourier transform infrared spectra of TCPP, PCN‐224 and PCN‐224/Fe; (E) BET sorption curve (the inset: hole size distribution of PCN‐224/Fe); (F) TG curve of CoNi‐MOF@PCN‐224/Fe.

X‐ray photoelectron spectroscopy (XPS) of the MOF‐on‐MOF structures was investigated, the results of which are shown in Figure 3A,B and Figure S5. The full XPS survey of CoNi‐MOF@PCN‐224/Fe indicates the existence of C, N, O, Zr, Fe, Co and Ni (Figure S5A). The result is consistent with the EDS analysis in Figure S4. For PCN‐224, the peak of Pyridinic N is centered at 396.3 eV. After the Fe^3+^ anchoring, the peak shifted positively to 396.7 eV due to the coordination between N and Fe^3+^ and the charge‐oriented transfer from N to Fe^3+^.^[^ ^32^ ^]^ This effect was also identified by fluorescence quenching of the system after the introduction of Fe^3+^ on account of the fluorescence inner filter effect (IFE) and Förster resonance energy transfer (FRET, Figure 3C and Figure S6). Moreover, the PCN‐224/Fe showed obvious Fe─N at the wavelength of 997 cm^−1^ and the N─H at 3310 cm^−1^ disappeared in comparison with PCN‐224 and TCPP, demonstrating the atom anchoring of Fe with PCN‐224 by Fe─N bonds (Figure 3D and Figure S2).^[^ ^32^ ^]^ The peak at 1050 cm^−1^ is attributed to deformation peak of pyrrole. After the formation of PCN‐224 and anchoring of Fe with PCN‐224 by Fe─N bonds, the variation of charge density distribution may cause a change in the dipole moment of C─N, which resulted in the increased peak at 1050 cm^−1^.^[^ ^31^ ^]^ Nitrogen sorption isotherms measured at 77 K (Figure 3E) display a typical type I isotherm with a Brunauer–Emmett–Teller (BET) surface area as high as 790.97 m^2^ g^−1^. The large surface area and well‐ordered porous structures of the nanozymes guarantee sufficient catalytic active sites and high‐flux mass transfer, which are essential to boost the catalytic reaction. After the assembly of CoNi‐MOF onto PCN‐224/Fe, high‐resolution XPS of Pyridinic N positively shifted (Figure 3A) while Fe 2p and Zr 3d peaks negatively shifted (Figure 3B and Figure S5B), suggesting strong interactions between PCN‐224/Fe and CoNi‐MOF and the successful preparation of CoNi‐MOF@PCN‐224/Fe nanozymes. Besides, the CoNi‐MOF@PCN‐224/Fe showed excellent thermal stability and the primary structure remained intact below 390°C (Figure 3F), which is beneficial for practical ECL detection applications.

### 2.3 Evaluation of the nanozyme‐enhanced ECL performance

**FIGURE 4:**
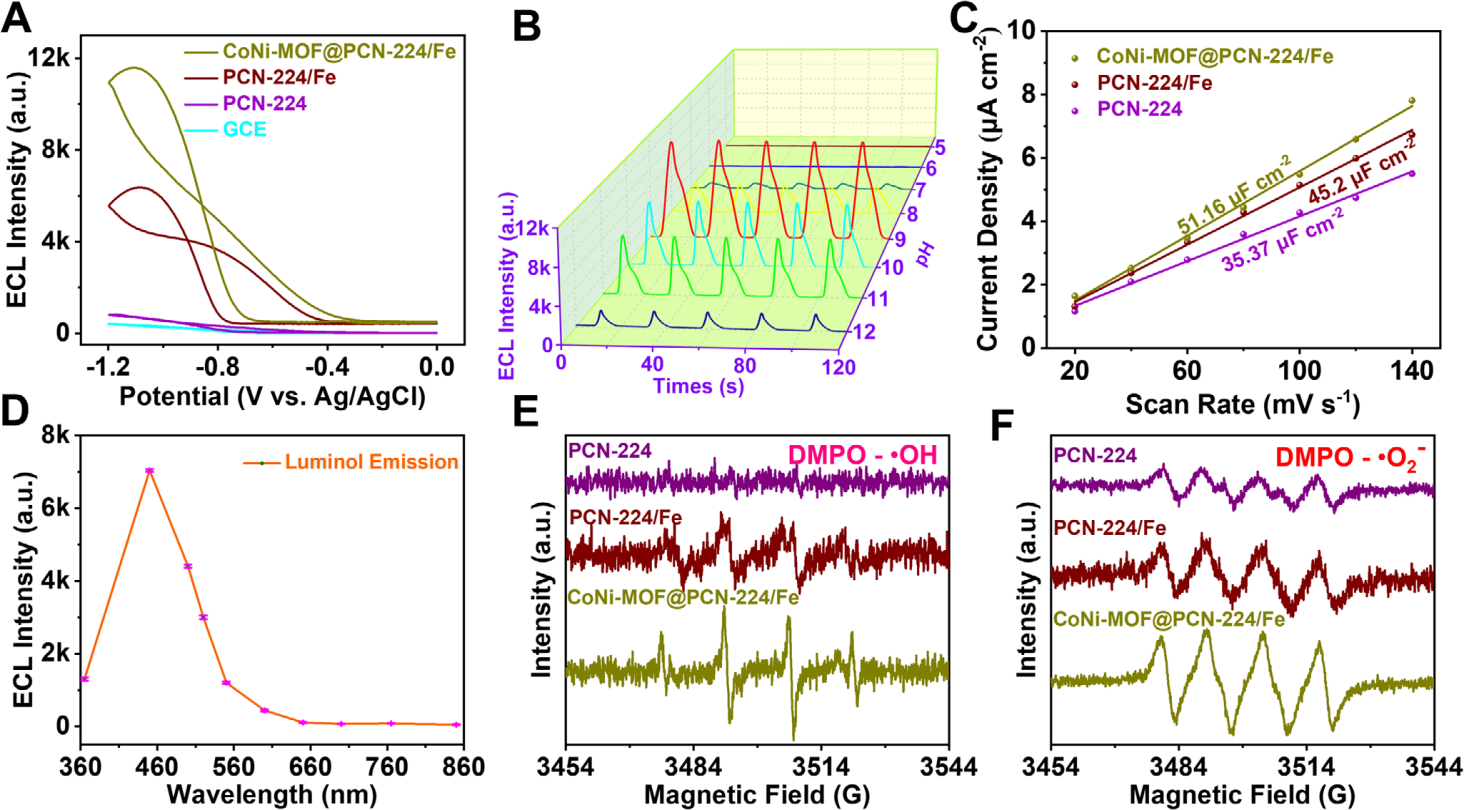
Investigation of the cathodic ECL performance. (A) ECL intensity of working glassy carbon electrode modified with PCN‐224, PCN‐224/Fe and CoNi‐MOF@PCN‐224/Fe, respectively, obtained in the solution of 10 μm H_2_O_2_ in PBS buffer (pH = 9), PMT 400 V; (B) ECL intensity of CoNi‐MOF@PCN‐224/Fe at different pHs; (C) The specific capacitance of PCN‐224, PCN‐224/Fe and CoNi‐MOF@PCN‐224/Fe, respectively; (D) ECL spectrum of luminol; EPR spectra of (E) DMPO‐·OH, and (F) DMPO‐·O_2_^−^ for PCN‐224, PCN‐224/Fe and CoNi‐MOF@PCN‐224/Fe.

We then investigated the cathodic ECL response and performance of the MOF‐on‐MOF nanozymes, the results of which are shown in Figure 4A,B and Figures S7, S8. The optimal pH and concentration of cocatalysts were set at 9.0 and 10 μmol L^−1^, respectively. This result is consistent with previous reports as luminol commonly exhibits the strongest luminescence efficiency at alkaline conditions.^[^ ^8^, ^13^, ^33^ ^]^ There was almost no ECL response on bare glassy carbon electrodes (GCE). Further modifications with the pristine PCN‐224 exhibited moderately enhanced ECL intensity due to the weak peroxidase‐like activity. However, once the iron atoms were anchored on PCN‐224, the cathodic ECL of PCN‐224/Fe was remarkably enhanced, demonstrating the high efficiency of the hemin‐like nanozymes in promoting ECL emission. This result is consistent with the DFT results that the atomically Fe act as the major catalytic activity sites and is capable of lowering the activation energy of the nanosystem to generate more ROS (Figure 1). After the in situ assembly of CoNi‐MOF, the cathodic ECL signal of the resulting nanosystem was further increased, which is about ≈12‐fold enhancement compared with the pristine PCN‐224 or control CoNi‐MOF, respectively (Figure 4A and Figure S9), proving the excellent ECL performance of CoNi‐MOF@PCN‐224/Fe. To calculate the electrochemically active surface area (*ECSA*) of the individual cathode, cyclic voltammetry (CV) curves with different scan rates were recorded to obtain the double‐layer capacitance (*C_dl_*). The comparison of *C_dl_* value of different samples displays that the CoNi‐MOF@PCN‐224/Fe had a larger *ECSA* (51.16 μF cm^−2^) than those of contrast cathodic materials (Figure 4C and Figures S10−12). The high active surface area was attributed to the unique lamellar surface morphology and hierarchical MOF‐on‐MOF nanostructures, which increase the contact area with the electrolyte and in accordance with the ECL results. The ECL spectra are shown in Figure 4D with an emission wavelength centered at ≈460 nm, which corresponded to the emission spectrum of the excited state oxidation product of luminol, demonstrating that the ECL signals were derived from luminol.^[^ ^33^ ^]^ Moreover, all the samples showed low ECL signals in nitrogen‐saturated luminol solution (without H_2_O_2_, Figure S13), suggesting the crucial role of H_2_O_2_ as a high‐efficient cocatalyst in the ECL system. The results indicate that the synergy of the Fenton‐like reaction of PCN‐224/Fe and CoNi‐MOF endows MOF‐on‐MOF nanozymes with higher peroxidase activity to produce ROS and hence the enhancement of ECL in the luminol‐H_2_O_2_ system. To prove this, electron paramagnetic resonance (EPR) was conducted as shown in Figure 4E,F and Figure S14. The characteristic peaks of DMPO‐•OH and DMPO‐•O_2_^−^ are pretty weak in the system containing PCN‐224 under light irradiation. Notably, obvious DMPO‐•OH and DMPO‐•O_2_^−^ signals were detected in the PCN‐225/Fe; Moreover, the intensities are further increased in the CoNi‐MOF@PCN‐224/Fe system, validating high peroxidase‐like catalytic activity of the CoNi‐MOF@PCN‐224/Fe nanozymes in ROS generation. Besides, isopropanol and benzoquinone were used as quenchers of •OH and •O_2_^−^ to identify the generation of •OH and •O_2_^−^ on CoNi‐MOF@PCN‐224/Fe. As shown in Figure S15, after the addition of isopropanol or benzoquinone, the ECL quenching was obviously observed, which is consistent with the EPR results and identified the peroxidase‐like property of CoNi‐MOF@PCN‐224/Fe. According to the standardized method for enzyme activity evaluation,^[^ ^34^ ^]^ the Michaelis–Menten constant (*K_m_*) and maximal reaction rate (*ν_max_*) were measured as displayed in Figure S16. The *K_m_* value of the nanozyme was calculated to be 1.23 mm for TMB with the *ν_max_* of 15.96 μm min^−1^, which is higher or comparable with other nanozymes reported (Table S1). On the basis of the above experimental results, a possible ECL mechanism of the luminol‐H_2_O_2_ system on CoNi‐MOF@PCN‐224/Fe catalysts is proposed as follows:

(1) $$Luminol\;\left(LH\right)-H^{+}\to L^{-}$$

(2) $$H_{2}O_{2}+e\;\overset{CoNi-MOFs@PCN-224/Fe}{\to }ROS$$

(3) $$ROS+L^{-}\to 3-aminophthalate{\;}^{2-*}\;$$

(4) $$\;3-aminophthalate{\;}^{2-*}\to 3-aminophthalate{\;}^{2-}+N_{2}+h\nu$$

According to the proposed mechanism, the luminol firstly deprotonates in an alkaline solution (Equation (1)) to generate L^−^ and the H_2_O_2_ was catalyzed by CoNi‐MOF@PCN‐224/Fe nanozymes to produce ROS (Equation (2)). Then the $L^{-}$ reacts with the electrogenerated ROS and forms the excited‐state intermediates (3‐aminophthalate^2‐*^). Finally, the excited 3‐aminophthalate^2‐*^ emits light as they return to the ground states (3‐aminophthalate^2‐^).

### 2.4 Construction of DSAS system and its application in PSA detection

**FIGURE 5:**
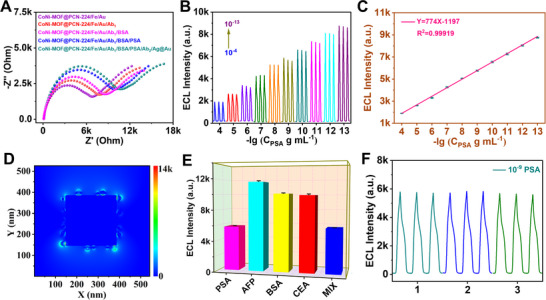
ECL performance for PSA detection. (A) EIS spectra of the samples in 20.0 mm K_3_[Fe(CN)_6_]/K_4_[Fe(CN)_6_] during sequential sample preparations; (B) ECL signals to PSA with different concentrations, and (C) corresponding calibration curve for PSA detection; (D) spatial EM field distribution of the Ag/AuNCs; (E) selectivity of the proposed ECL immunosensor for PSA detection; (F) reproducibility of the immunoassay with 10^−9^ g mL^−1^ of PSA.

As shown in Scheme 1, the differential signal amplification strategy (DSAS) based ECL system was established by using Ag/AuNCs as high‐efficiency ECL label quenchers and PSA as a model disease biomarker. In the presence of PSA, the Ab_2_‐labeled Ag/AuNCs will bond to the nanozyme decorated ECL electrode specifically. In the luminol‐H_2_O_2_ ECL system, the more Ag/AuNCs bound, the faster the H_2_O_2_ will be consumed by etching Ag nanocubes.^[^ ^35^ ^]^ Thus, the ECL signal is inversely proportional to the concentration of PSA in the test solution. Figure S17 shows the SEM and TEM images of the label quencher (Ag/AuNCs) which exhibited uniform nanocubes with a side length of ≈150 nm. Then, the DSAS‐based ECL system for immunoassay application was checked by using PSA as a target. As shown in Figure 5A, the resistance of the system decreased gradually with the modification of antibodies due to the insulating property of the biomolecular, which confirmed the successful preparation of the sandwich‐type ECL immunoassays.^[^ ^36^ ^]^ The ECL intensity reduced with the increase of PSA content, which displays a good linear relationship between the PSA concentration and ECL signal in the range from 10^−4^ to 10^−13^ g mL^−1^ (Figure 5B,C). The sensitivity and limit of detection (LOD) are calculated to be 7740 mol^−1^ L and 0.13 pg mL^−1^, respectively. The LOD is lower or comparable with those previously reported (Table S2). The ultrasensitive detection ability of the ECL system is attributed to the favorable catalytic kinetics and thermodynamic behavior of the CoNi‐MOF@PCN‐224/Fe nanozyme and the high‐efficiency ECL quencher of Ag/AuNCs. When Ab_2_‐labeled Ag/Au nanocubes are presented in the luminol‐H_2_O_2_ system, the cocatalyst (H_2_O_2_) will be directly consumed by etching the Ag/AuNCs (Figure S18). Moreover, the absorption spectrum of Ag/AuNCs overlaps with the emission spectrum of luminol (Figure S19), and the subsequent surface plasmon coupling can generate enhanced electromagnetic field hot spots around Ag/AuNCs (Figure 5D) to accelerate the depletion of H_2_O_2_ as identified by the photoelectrochemical H_2_O_2_ decomposition experiments shown in Figure S20.^[^ ^37^ ^]^ By contrast, the ECL cathode only displayed a sensitivity of 4980 mol^−1^ L while using traditional reducing agents (ascorbic acid, AA) as H_2_O_2_ consumption agents, suggesting the superiority of the designed Ag/AuNCs ECL quencher (Figure S21). To investigate the specificity of the immunoassays for PSA detection, several antigen analogs containing AFP, CEA, BSA were employed to assess the detection selectivity (Figure 5E). No obvious ECL signal changes were observed when the detection targets were AFP, CEA and BSA (10^−8^ g mL^−1^), while for PSA and the mixture containing PSA and the above interferents, the ECL intensity decreases remarkably, which indicates the excellent specificity of the DSAS‐based immunoassay for PSA. Moreover, the immunoassay exhibits good repeatability as displayed in Figure 5F. These results suggested that the developed DSAS‐based immunoassay was very suitable for the ultrasensitive detection of PSA with excellent sensitivity, selectivity, and stability. Finally, the real application of the immunosensor was assessed by detecting the concentration of PSA in human serum. As shown in Table S3, the developed immunosensors showed good recovery, suggesting the validity of the DSAS‐based ECL immunoassays in the detection of disease biomarkers. Moreover, the immunoassay exhibited good detection performance in detecting real clinical samples (276.3 ng mL^−1^), consistent with the result by using ELISA kit (251.8 ng mL^−1^), with an error of 9.7%.

## 3 CONCLUSION

In summary, atomically Fe‐anchored MOF‐on‐MOF nanozymes (CoNi‐MOF@PCN‐224/Fe) were designed and exploited as high‐efficiency ECL electrode materials in the luminol‐H_2_O_2_ system. Mechanistic insights proved that the remarkably enhanced ECL was attributed to the Fenton‐like catalytic reaction and multi‐metal‐sites of the CoNi‐MOF@PCN‐224/Fe, which obviously decrease the Gibbs free energy of the reaction and promote the generation of the reactive oxygen species. Moreover, a novel signal amplification strategy, DSAS, was proposed and successfully developed by designing novel plasmonic Ag/AuNCs as high‐efficient co‐reactant quenchers in the ECL immunoassay. The developed nanozyme immunoassay exhibited excellent detection ability for PSA with an LOD of 0.13 pg mL^−1^. The proposed signal amplification strategy opens a new pathway in designing high‐efficiency ECL sensing platforms, which could be widely used in biomedical and chemical analysis, especially in the ultrasensitive detection of target disease biomarkers in clinical diagnosis.

## CONFLICT OF INTEREST STATEMENT

The authors declare no conflicts of interest.

## Supporting information

Supporting information

## Data Availability

All data of this work are present in the article and Supporting Information. The other data that support the findings of this work are available from the corresponding author upon reasonable request.
