# Supplementary material for: Atomically Fe‐anchored MOF‐on‐MOF nanozyme with differential signal amplification for ultrasensitive cathodic electrochemiluminescence immunoassay
Source: Exploration (Beijing). 2023 Jul 7;3(4):20220151. doi: 10.1002/EXP.20220151 (PMC10624370; doi:10.1002/EXP.20220151)
Supplement: Supplementary file 1 — Supporting information [file EXP2-3-20220151-s001.docx]

Supporting Information

Atomically Fe Anchored MOF-on-MOF Nanozyme with Differential Signal Amplification for Ultrasensitive Cathodic Electrochemiluminescence Immunoassay

Chuanping Li,^*^ Tianxiang Hang, Yongdong Jin^*^

**Experimental Procedures**

**Chemicals and Materials**

Ethanol, methanol, 2-methylimidazole (2-MIM), AgNO_3_, H_2_O_2_, ZrOCl_2_·8H_2_O and FeCl_3_ were purchased from Sinopharm Reagent Co. Tetrakis(4-carboxyphenyl)porphyrin (TCPP) was obtained from TCI Co., Ltd. Acetic acid (HAc), HAuCl_4_, Polyvinyl Pyrrolidone (PVP, M_w_=58000), 3,3',5,5'-Tetramethylbenzidine (TMB), Nafion (5%), NaBH_4_, Ni(NO_3_)_2_·6H_2_O, Co(NO_3_)_2_·6H_2_O, K_2_CO_3_, Na_2_HPO_4_, NaH_2_PO_4_, K_3_[Fe(CN)_6_] and K_2_[Fe(CN)_6_] were purchased from Aladdin Reagent Company (Shanghai, China). Carcino-embryonic antigen (CEA), AFP, PSA, Anti-KLK_3_ mouse monoclonal antibody (Ab_1_) and Anti-KLK_3_ rabbit polyclonal antibody (Ab_2_) were purchased from Sangon Biotech.

**Synthesis of PCN-224**

The PCN-224 nanocubes (NCs) were synthesized by a facile hydrothermal method. Firstly, 125 mg of ZrOCl_2_·8H_2_O was added to the 50 mL of DMF solvent. Subsequently, 25 mg of TCPP was added to the solvent and kept stirred for another ten minutes. After that, 12.5 mL of acetic acid was added to the mixed solution. Then, the solution was put into teflon autoclave and heated at 393.15 K for 12 hours. The generated precipitate was collected by centrifugation and dried under vacuum at 80 °C.

**Synthesis of PCN-224/Fe**

PCN-224/Fe was synthesized based on a wet chemical method. 40 mg of FeCl_3_ and 30 mg of PCN-224 NCs were added into 15 mL of DMF and stirred for half an hour. Then, the purple solution was heated at 120 °C for 1 h with slow magnetic stirring. After the reaction, rust color PCN-224/Fe NCs were obtained.

**Synthesis of PVP coated PCN-224/Fe**

Typically, the as-prepared PCN-224/Fe NCs were dispersed into 30 mL of PVP (0.1g mL^-1^) methanol solution for 24 h. After washing with centrifugation, PVP-coated PCN-224/Fe NCs were dispersed into methanol (30 mL) for use.

**Synthesis of CoNi-MOF and CoNi-MOF@PCN-224/Fe**

The lamellate CoNi-MOF was synthesized as follows. Firstly, 0.2463 g of 2-MIM was dissolved in methanol (10 mL) to form solution A. Then, 0.2908 g of Ni(NO_3_)_2_·6H_2_O and 2.6193 g of Co(NO_3_)_2_·6H_2_O were dispersed in methanol (30 mL) to form solution B. Finally, mixed the two solutions and stirred for 1 minute, then kept static at room temperature for 12 h. After that, the solution was washed with 25 mL of methanol three times. CoNi-MOF@PCN-224/Fe was successfully fabricated by replacing the 30 mL of methanol in B solution with the redispersed fresh methanol containing PCN-224/Fe/PVP.

**Synthesis of AgNCs and AgAuNCs**

5 mL of EG was added in a round-bottomed flask and heated at 140 ℃ for 1 h. Next, 1 mL of HCl (3 mmol in EG) was added. 10 min later, 3 mL of AgNO_3_ (94 mmol in EG) and 3 mL of PVP (147 mmol in EG) were synchronously added with a rate of 0.75 mL/min. The flask was further heated at 140 ℃ for 22 h. AgNCs were obtained by washing with acetone and water. AgAuNCs were obtained by using an ion exchange reaction. 0.01% of methanol solution of HAuCl_4_ was dropwise added into the stirred AgNCs aqueous solution until the color turned to olive green.

**Construction of the immunoassays**

(1) Preparation of Ab_1_ decorated CoNi-MOF@PCN-224/Fe electrode.

Firstly, 10 mg of CoNi-MOF@PCN-224/Fe was dispersed in 10 mL of methanol and 600 μL of HAuCl_4_ (1%). Then 1.2 mL of NaBH_4_ (0.1 M) was added drop-by-drop to obtain the AuNPs-decorated CoNi-MOF@PCN-224/Fe. After that, 8 μL of AuNPs-decorated CoNi-MOF@PCN-224/Fe (10 mg mL^-1^) was dropped on the glassy carbon electrode. Then, Ab_1_ (10 μL, 10^-4^ g mL^-1^) was added and incubated overnight at 4 ℃. With the strong bonding capability between the amino group of the antibodies and the AuNPs of CoNi-MOF@PCN-224/Fe, the antibodies could be effectively labeled on the nanomaterials via simply mixing. Next, PBS buffer (0.01 M) was used to remove the non-specific bonded Ab_1_. The fresh antibody was blocked by using 1% BSA. Finally, PSA antigen with different concentrations (10 μL) were added on the GC electrode and incubated for 2 h, followed by washing with 0.01 M PBS solution (pH=7.4).

(2) Preparation of AgAu-Ab_2_ bioconjugates

0.2 mg/mL of Ab_2_ (20 µL) was added into AgAuNCs (2.0 mL) and stirred for 4 h. Ultimately, 10 μL of AgAu-Ab_2_ bioconjugates were added to the GC electrode and incubated for 1 h, followed by washing with PBS solution (0.1 M, pH=7.4).

**ECL measurements**

The working electrode is the modified GC electrode, the reference electrode is Ag/AgCl electrode, and the counter electrode is the platinum sheet. The ECL test was carried out in 20 mL of PBS solution (0.1 M, pH 7.4) containing 100 μM luminol and 10 μM H_2_O_2_, PMT was set at 500 V.

**Structure and Property Characterizations of the Materials**

The morphology was characterized by a scanning electron microscope (Hitachi S4800) and transmission electron microscopy (FEI TalosF200x). X-Ray Diffraction (XRD) was performed via a D8 ADVANCE instrument (Bruker, Germany). EPR was performed by using an EPR spectrometer (Bruker EMXPLUS). The X-ray photoelectron spectroscopy (XPS) was characterized by using an XPS analysis (Thermo ESCALAB 250). UV-Vis diffuse reflectance spectrum (DRS) was performed on a spectrophotometer (Shimadzu UV-3600). The LOD was calculated on the basis of the following equations (S/N= 3) .

I=-774*logc (g mL^-1^)-1197


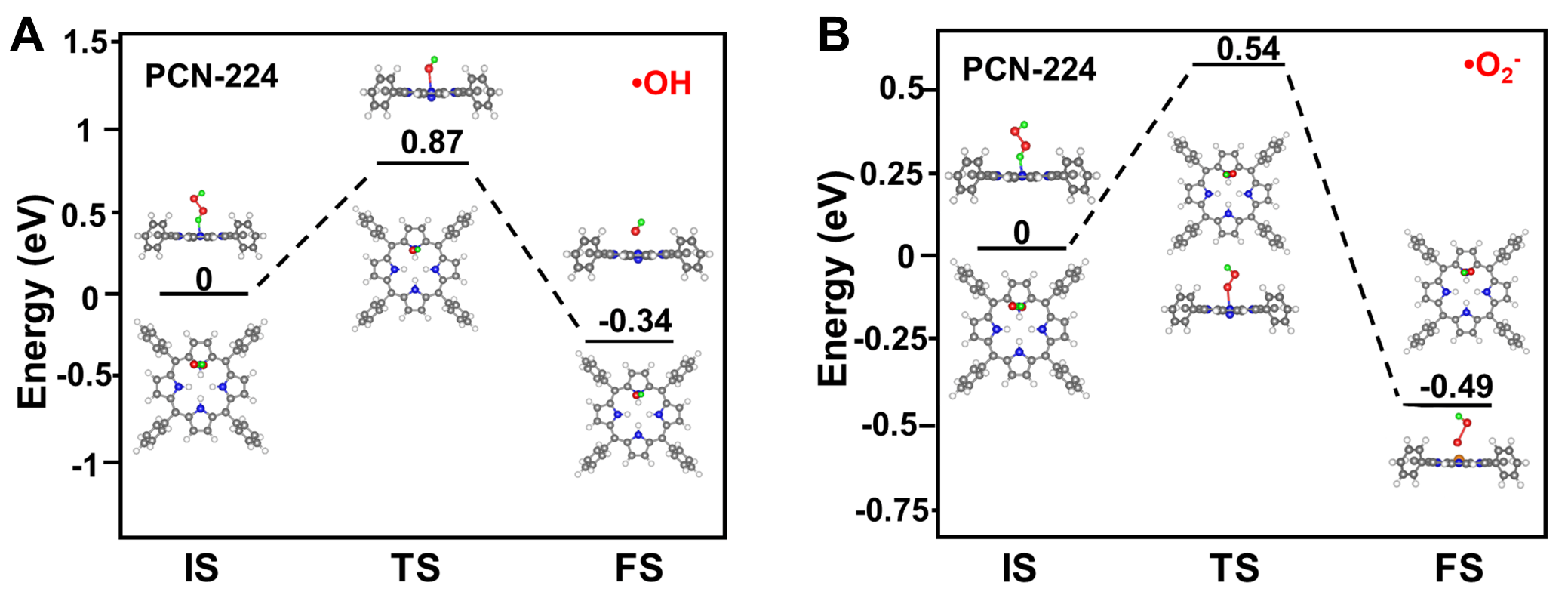


**Figure S1.** Reaction mechanism diagram and free energy scheme of the generation of •OH (A) and (B) •O_2_^-^ on PCN-224.


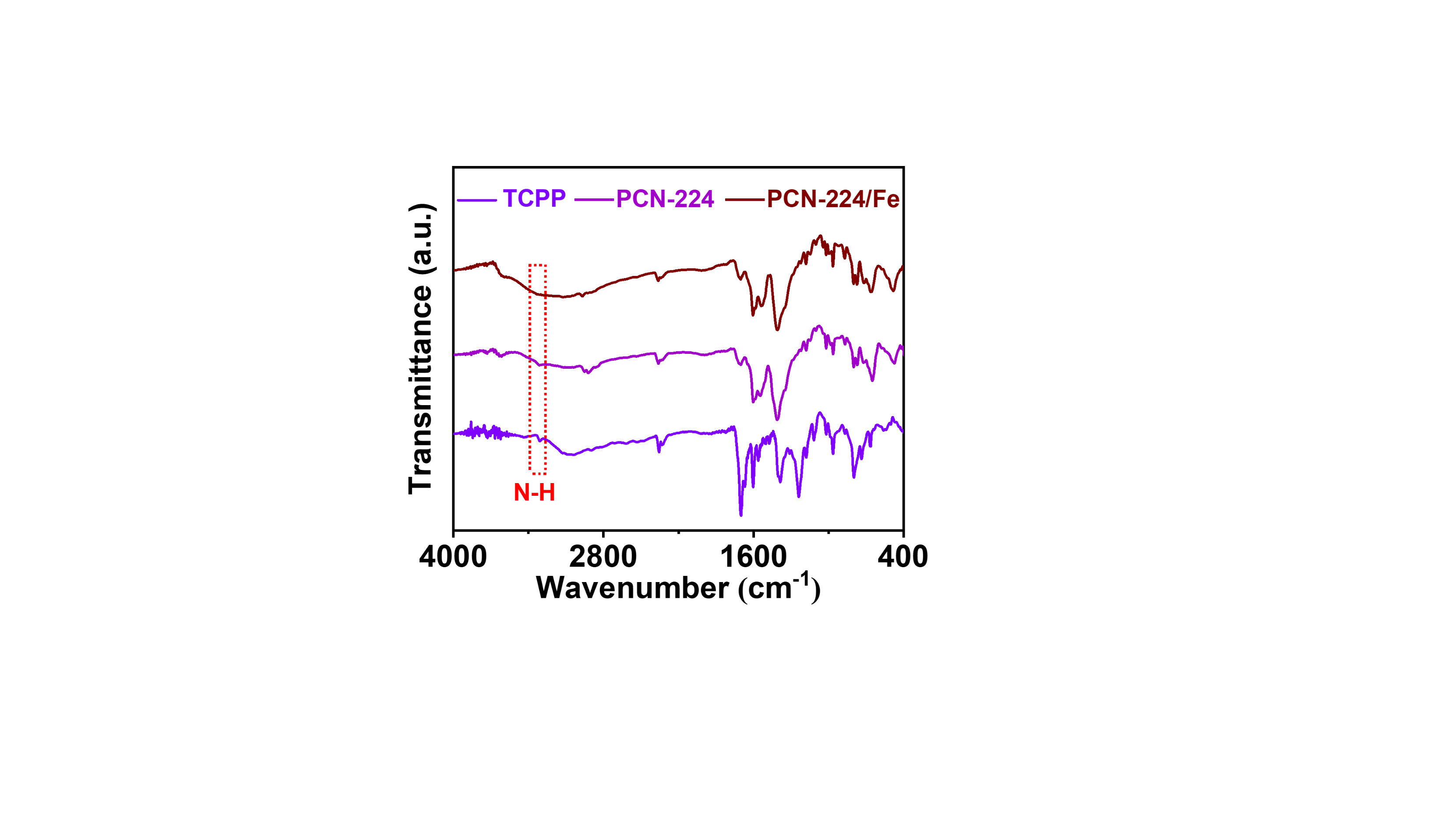


**Figure S2.** FTIR of TCPP, PCN-224 and PCN-224/Fe.


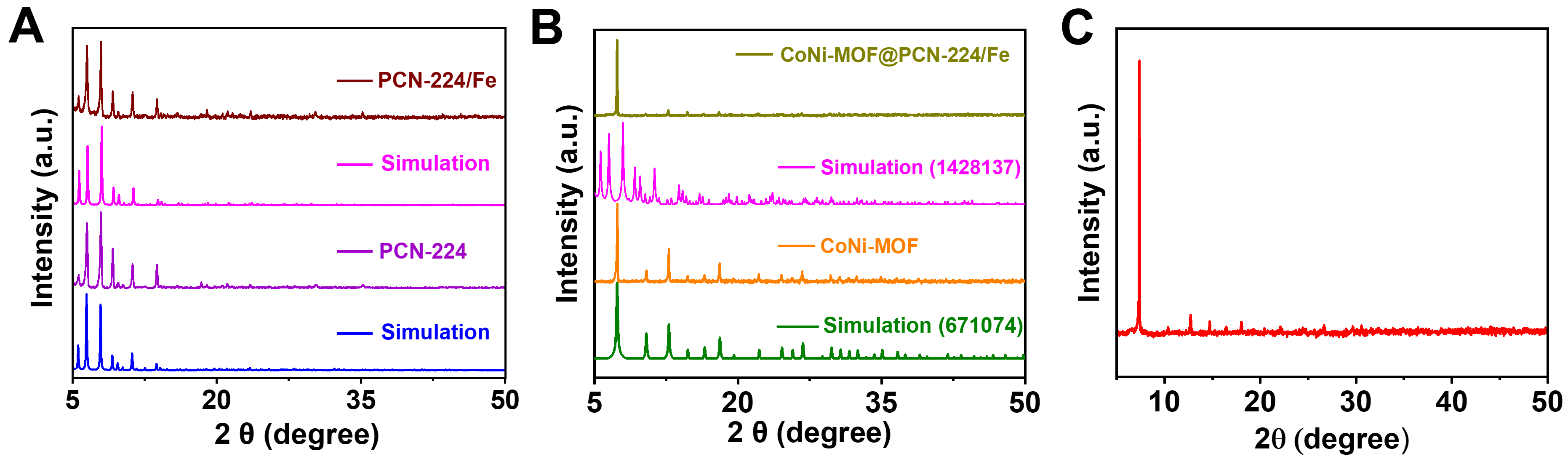


**Figure S3.** PXRD pattern of (A) PCN-224, PCN-224/Fe and (B) CoNi-MOF, CoNi-MOF@PCN-224/Fe, (C) Enlarged PXRD pattern of CoNi-MOF.


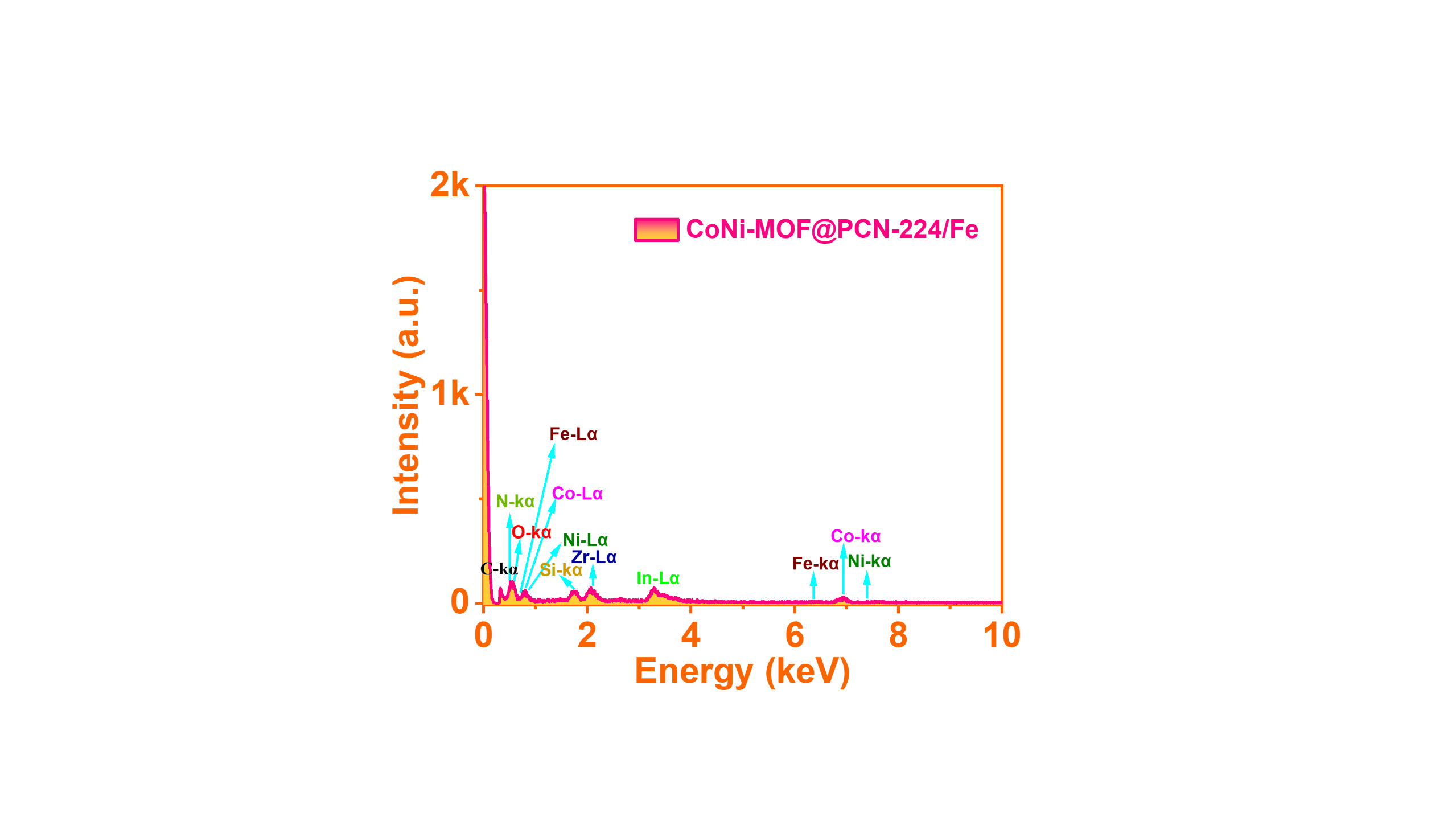


**Figure S4.** EDS analysis of CoNi-MOF@PCN-224/Fe.


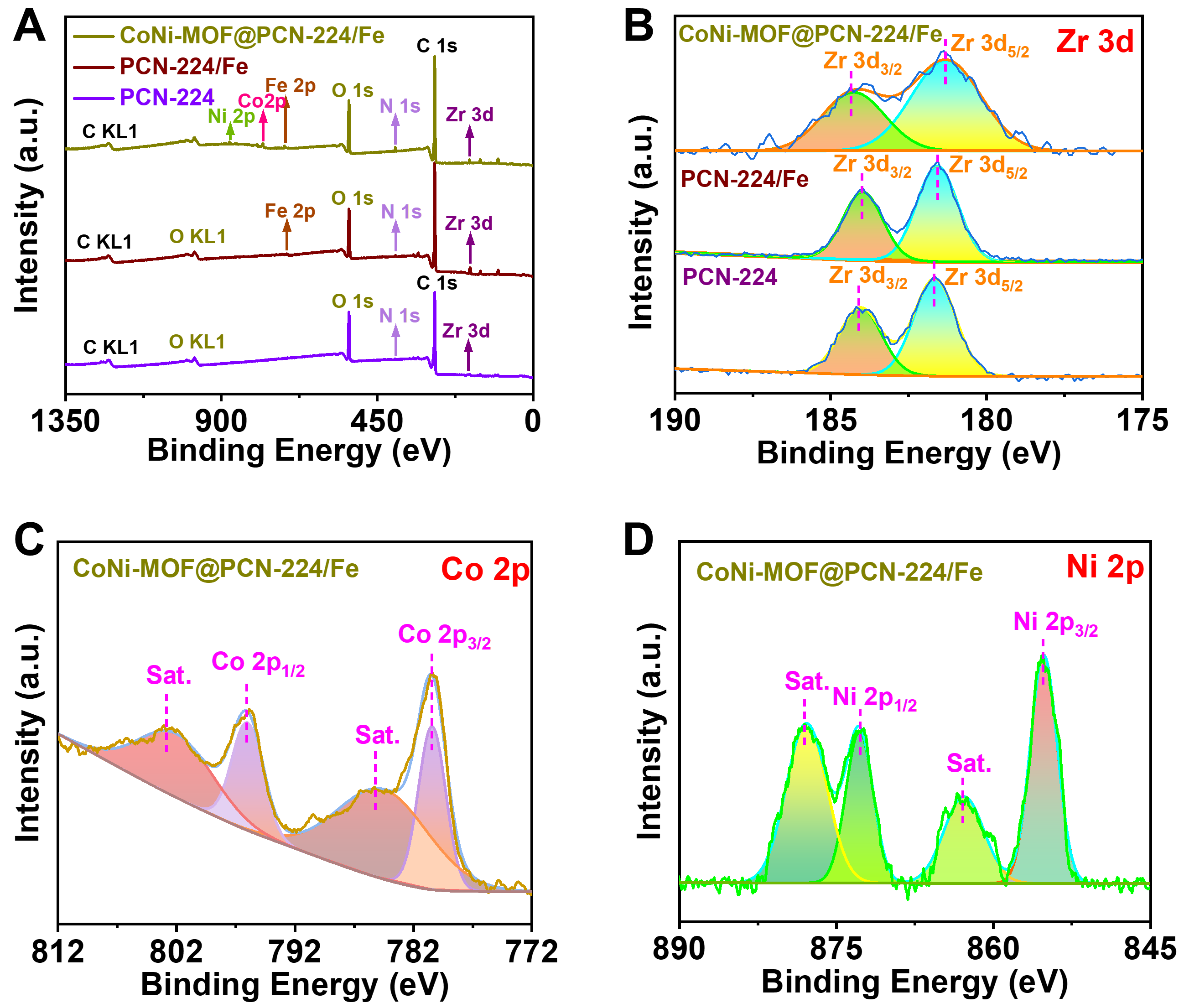


**Figure S5.** (A) Full-scan XPS spectra of PCN-224, PCN-224/Fe and CoNi-MOF@PCN-224/Fe, respectively. (B-D) High-resolution XPS spectra of (B) Zr 3d, (C) Co 2p and (D) Ni 2p.


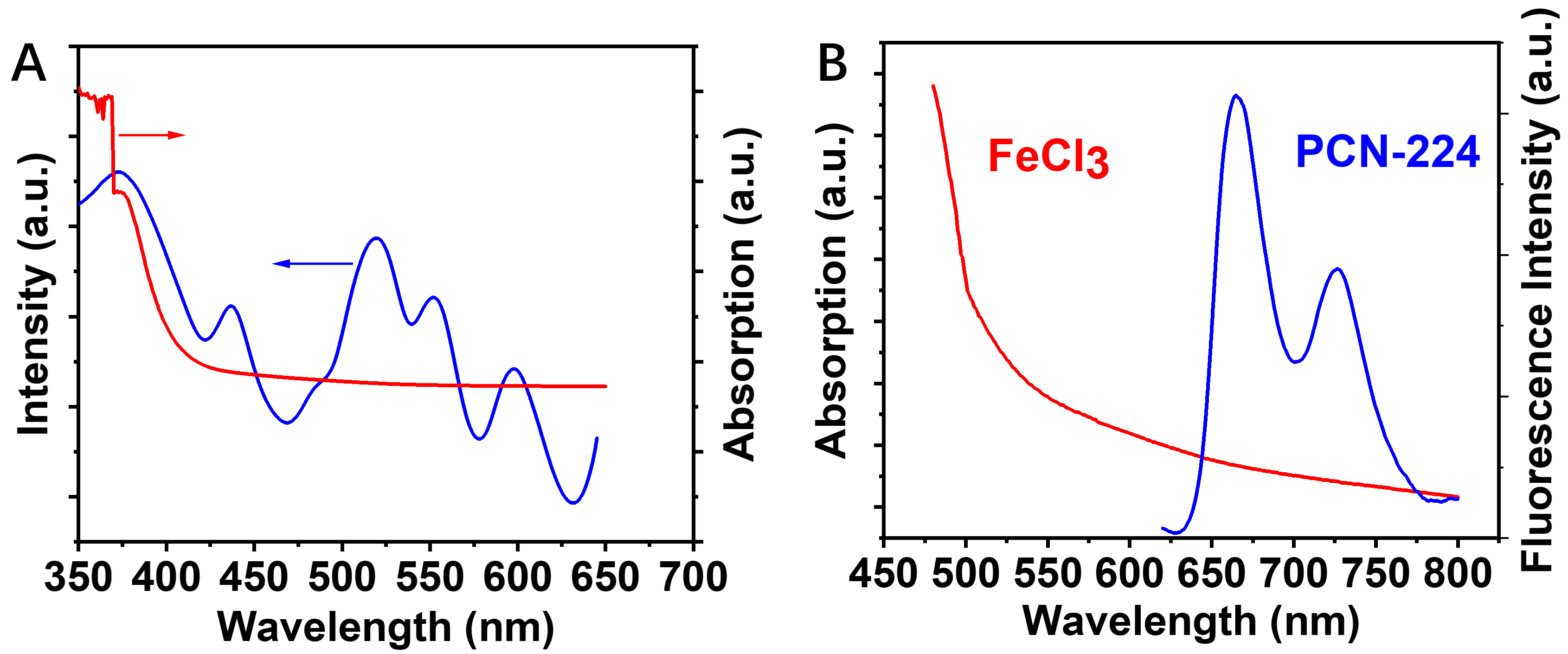


**Figure S6.** (A) The UV-Vis spectrum of FeCl_3_ and excitation spectrum of PCN-224. (B) The UV-Vis spectrum of FeCl_3_ and fluorescence spectrum of PCN-224.


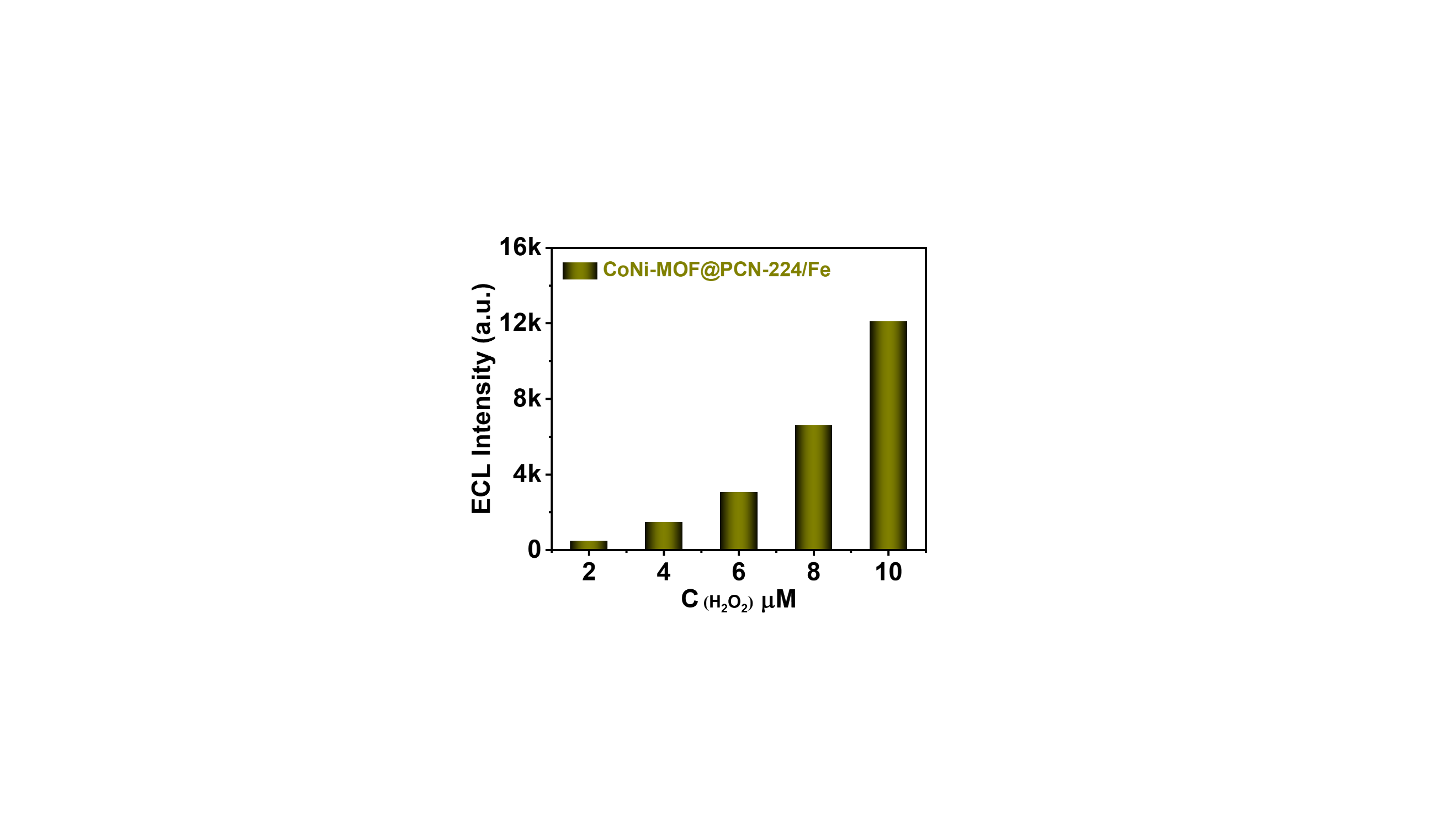
**Figure S7.** The ECL intensity of CoNi-MOF@PCN-224/Fe with different concentrations of H_2_O_2_.

**
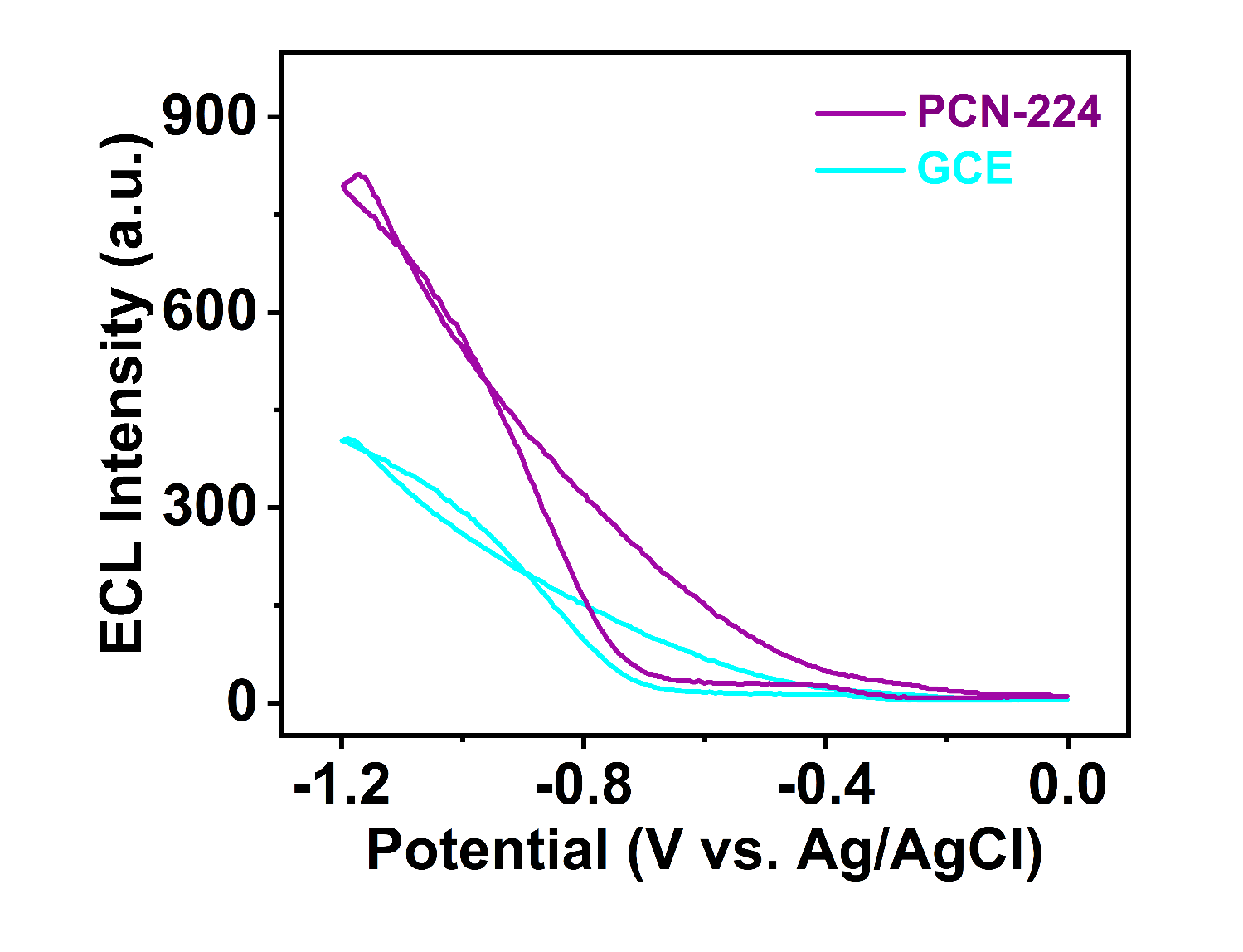
Figure S8.** Enlarged ECL intensity of GC and PCN-224 obtained in the solution of 10 μM H_2_O_2_ in PBS buffer (pH=9), PMT 400V.


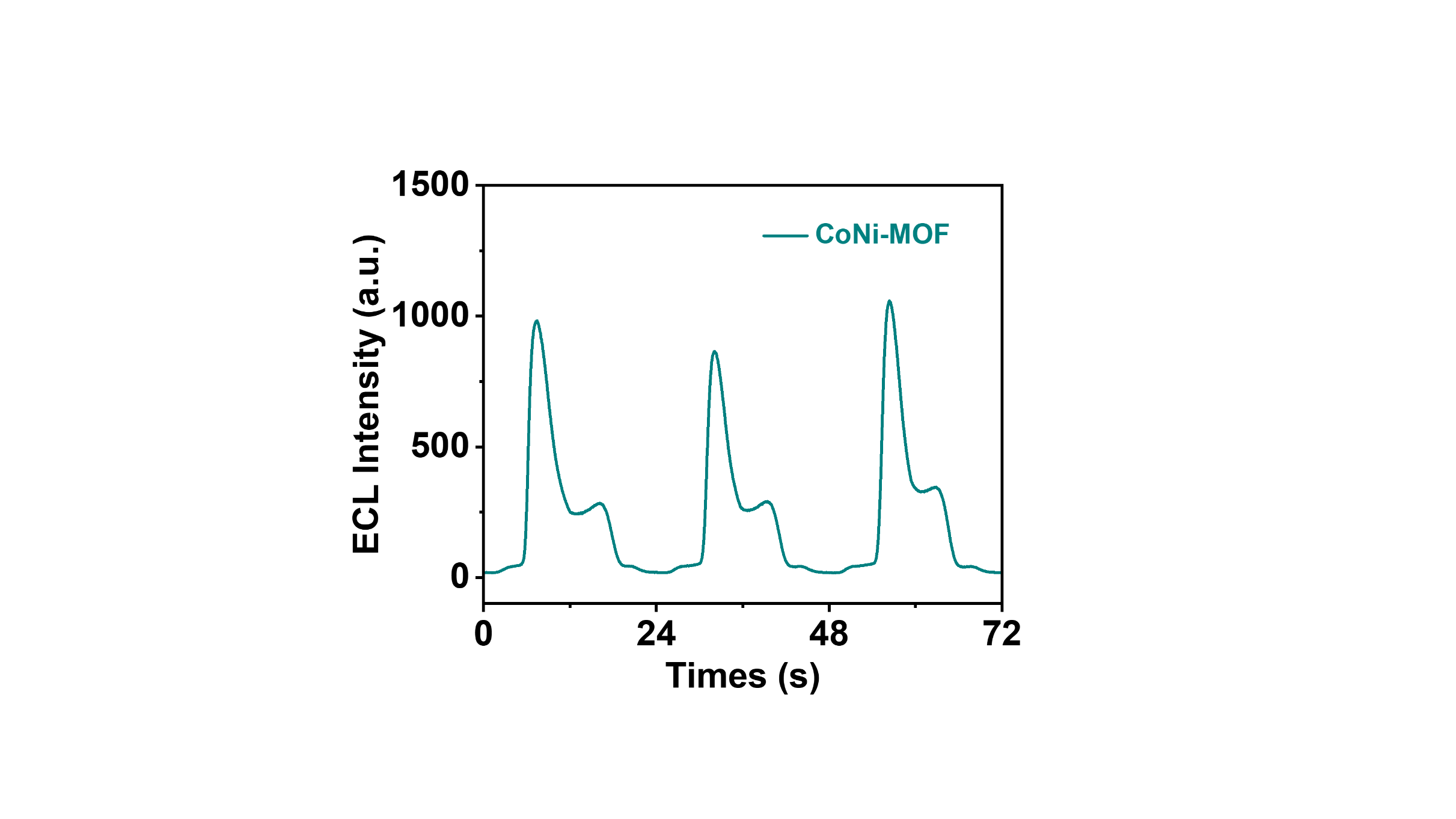


**Figure S9.** The cathodic ECL of CoNi-MOF.


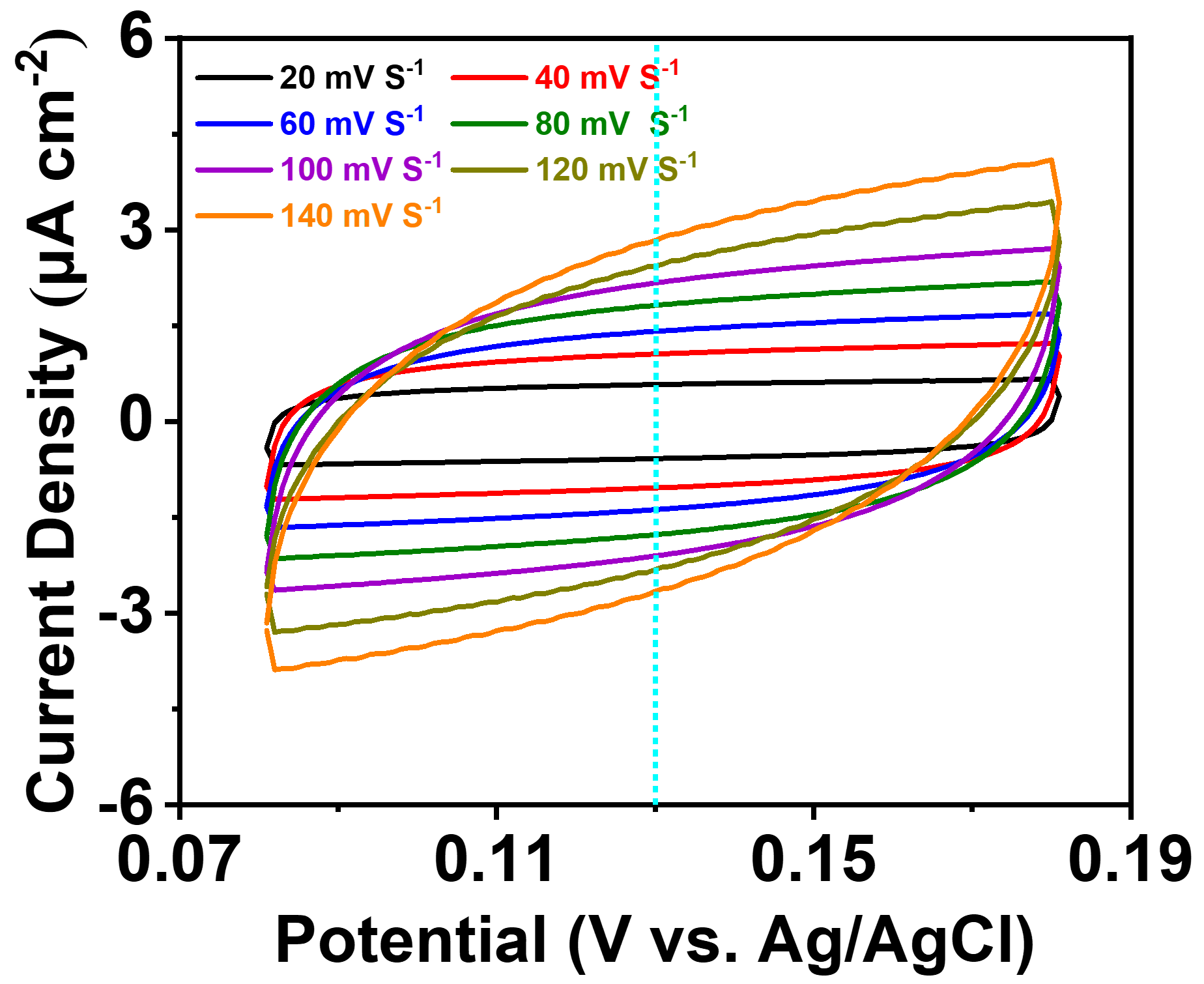


**Figure S10.** Cyclic voltammogram (CV) of PCN-224 recorded in 0.15 M KPF_6_/CH_3_CN.


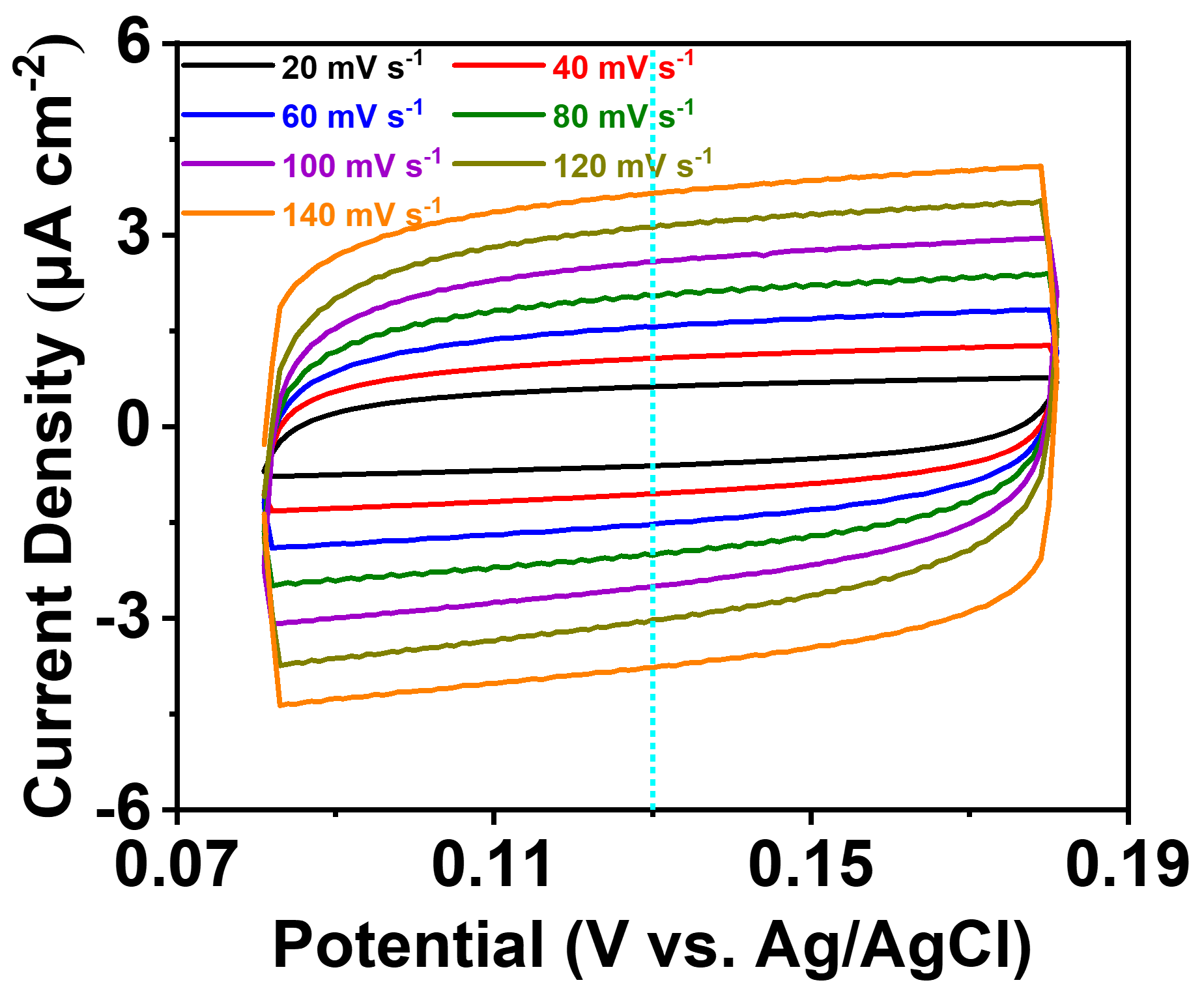


**Figure S11.** Cyclic voltammogram of PCN-224/Fe recorded in 0.15 M KPF_6_/CH_3_CN.


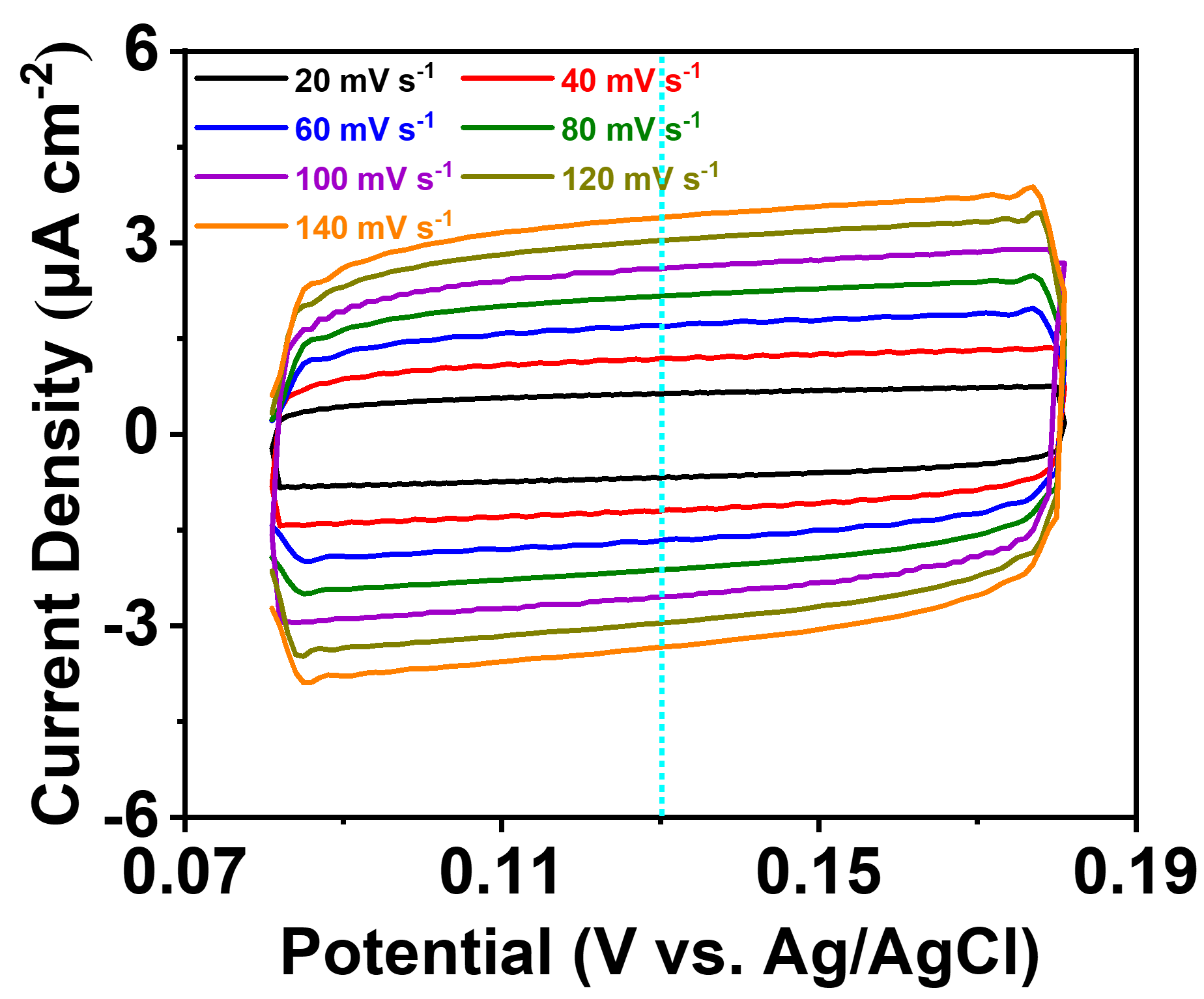


**Figure S12.** Cyclic voltammogram of CoNi-MOF@PCN-224/Fe recorded in 0.15 M KPF_6_/CH_3_CN.


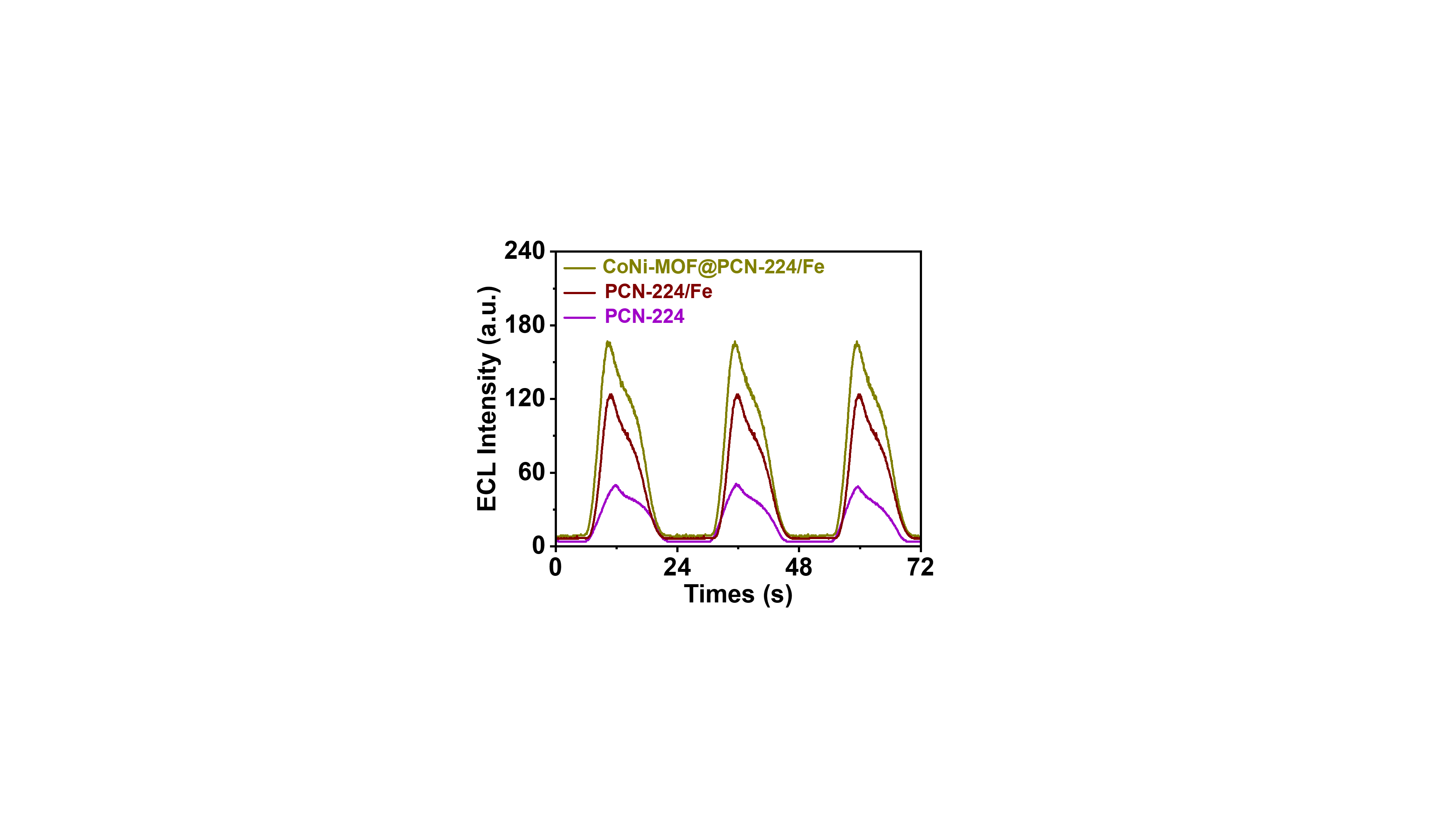


**Figure S13.** ECL of PCN-224, PCN-224/Fe and CoNi-MOF@PCN-224/Fe, respectively, without using H_2_O_2_ as cocatalyst.


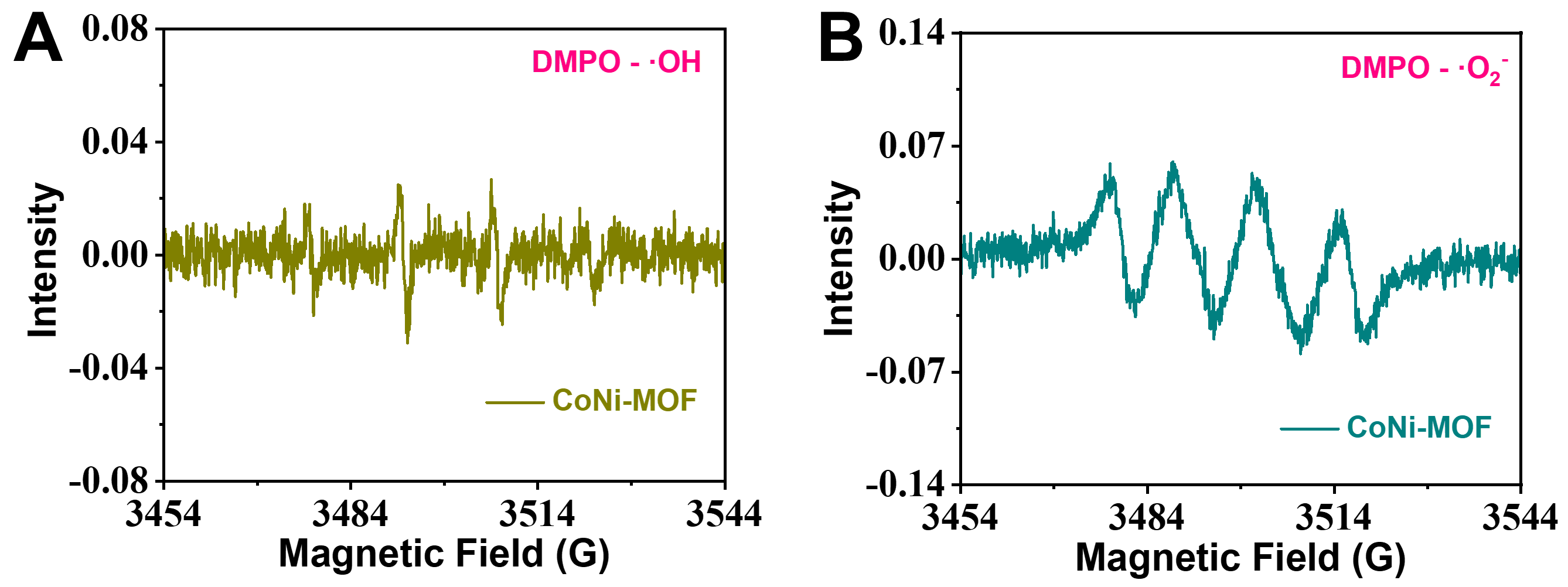


**Figure S14.** EPR spectra of (A) DMPO-·OH, and (B) DMPO-·O_2_^-^ for CoNi-MOF.


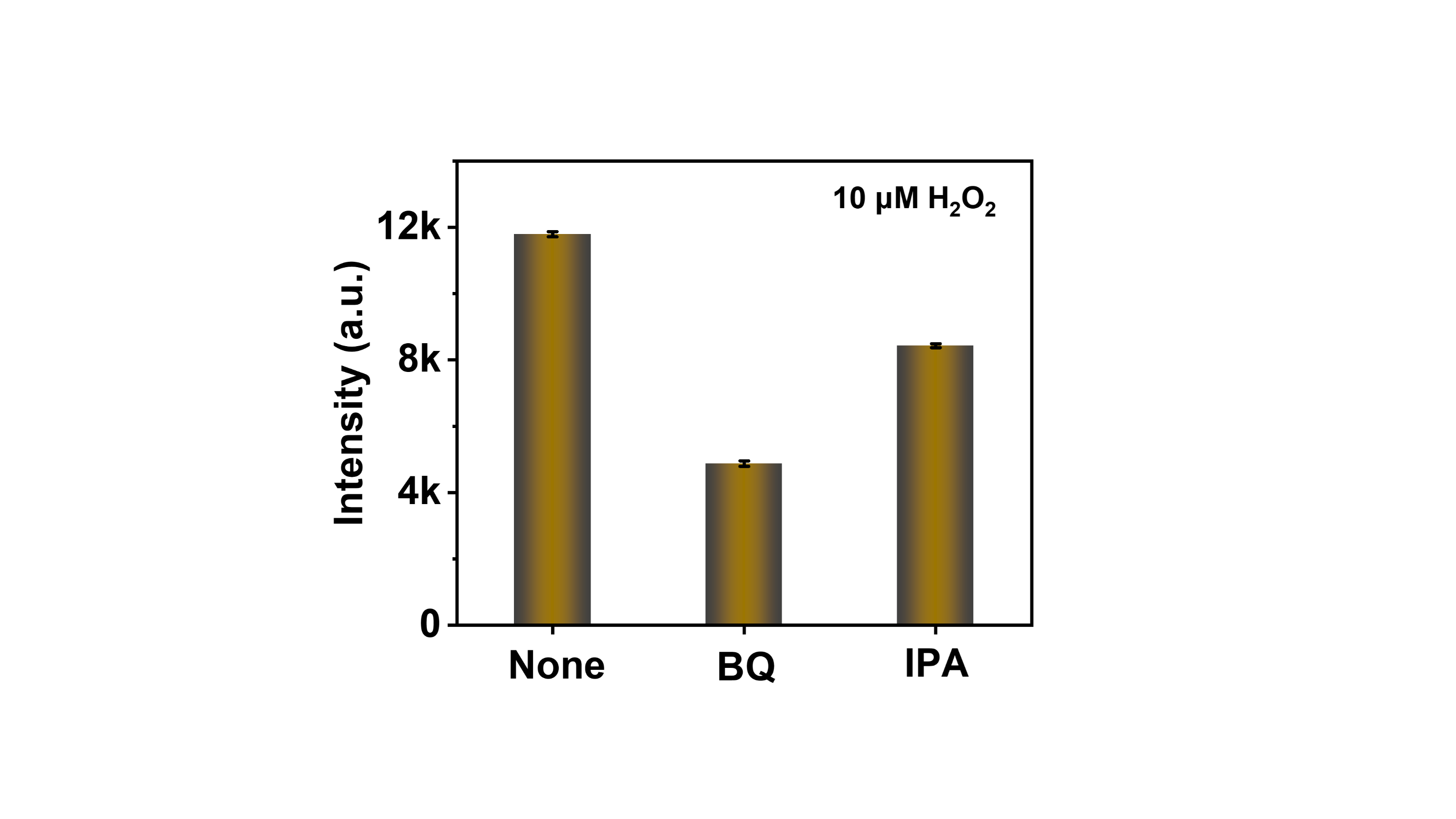


**Figure S15.** ECL intensity graph of CoNi-MOF@PCN-224/Fe with the addition of different radical scavengers.


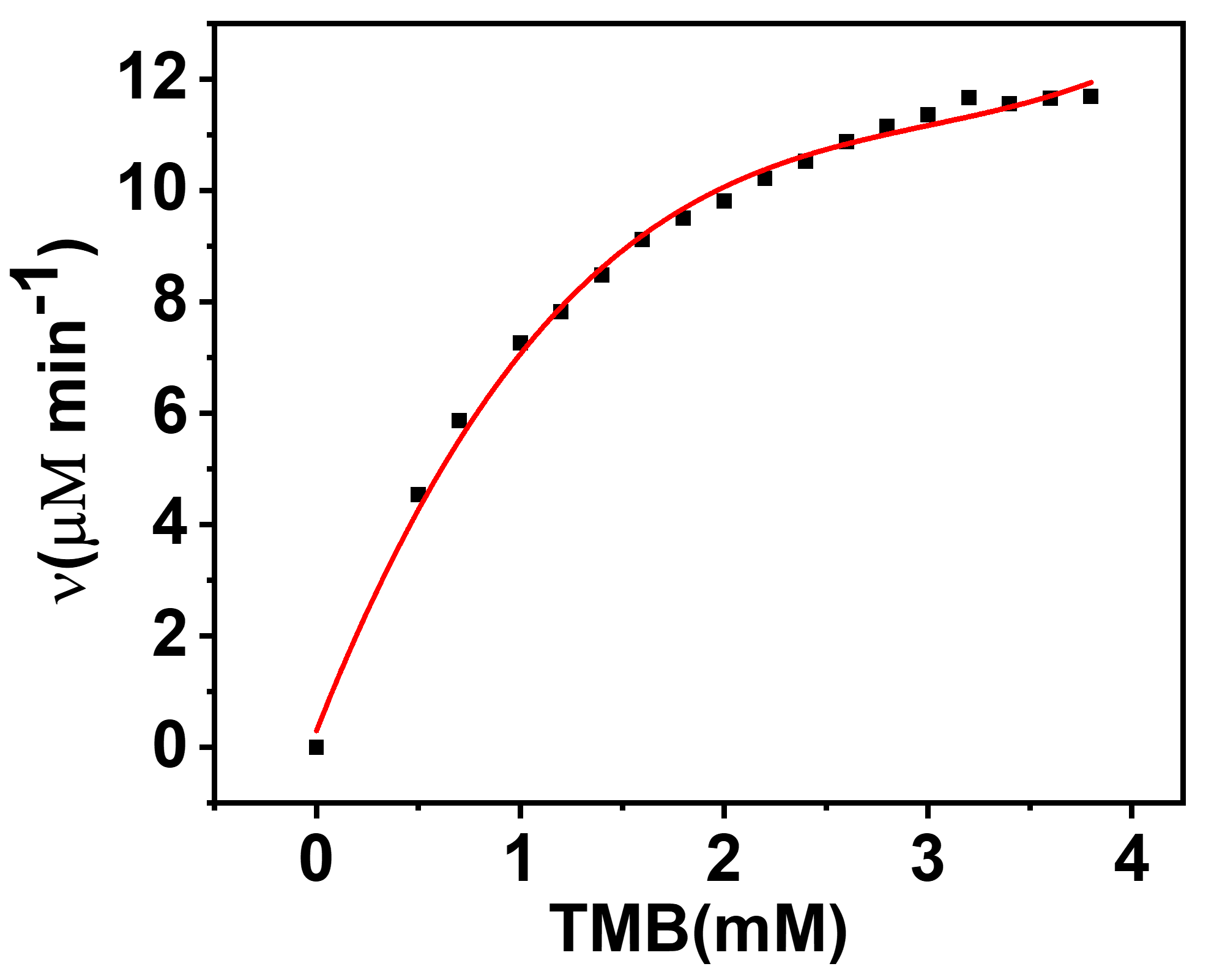


**Figure S16.** Michaelis–Menten curve for the tested CoNi-MOF@PCN-224/Fe nanozyme.


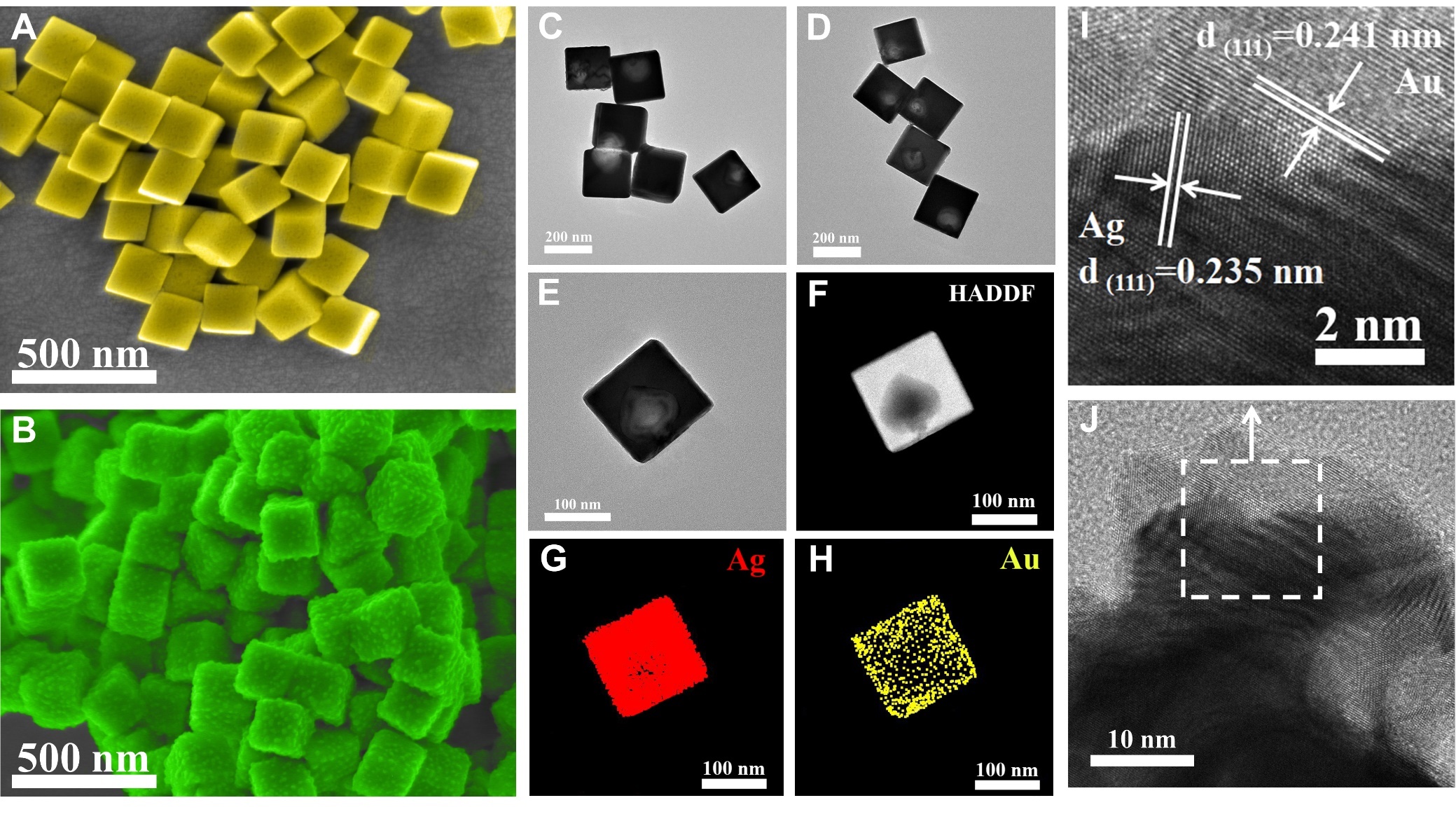


**Figure S17.** SEM images of (A) AgNCs and (B) AgAuNCs, and (C-E) TEM images, (F) HAADF, (G-H) elemental mapping, and (I-J) high-resolution TEM images of AgAuNCs.


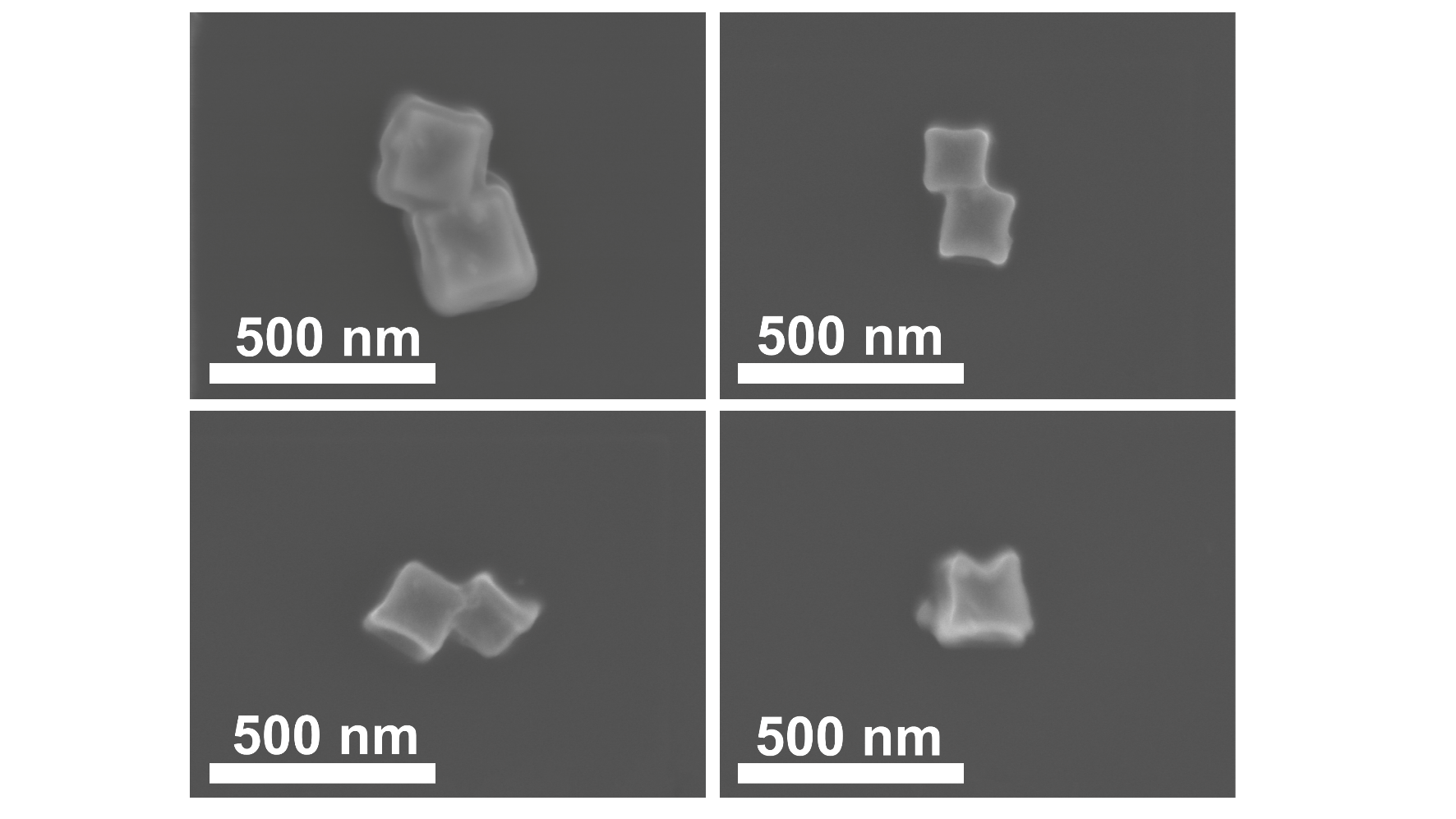


**Figure S18.** SEM images of Ag/AuNCs after the PSA detection in the luminol-H_2_O_2_ (10 μmol/L) solution.


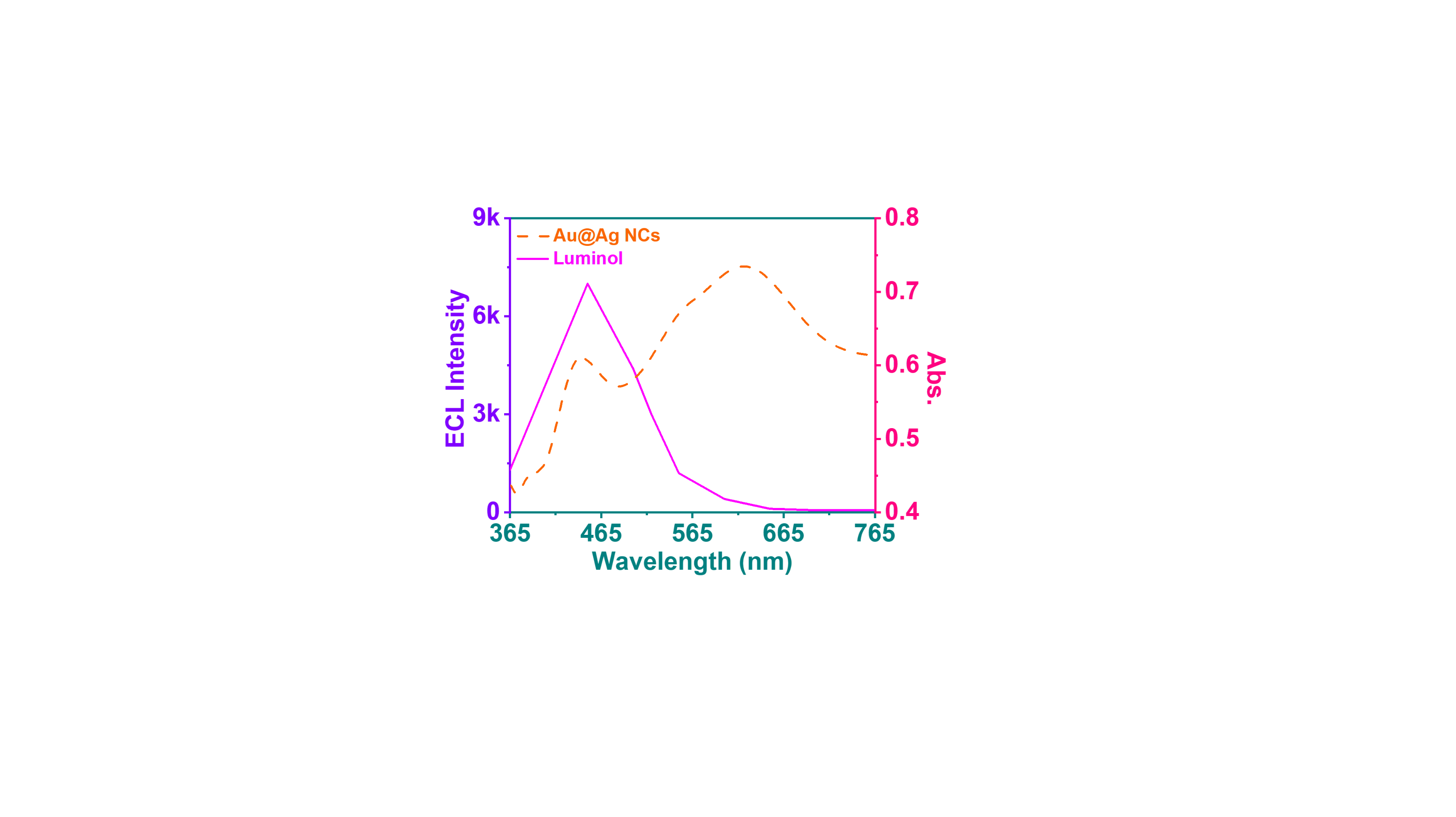


**Figure S19.** The emission spectrum of luminol and UV-Vis spectrum of AgAuNCs.


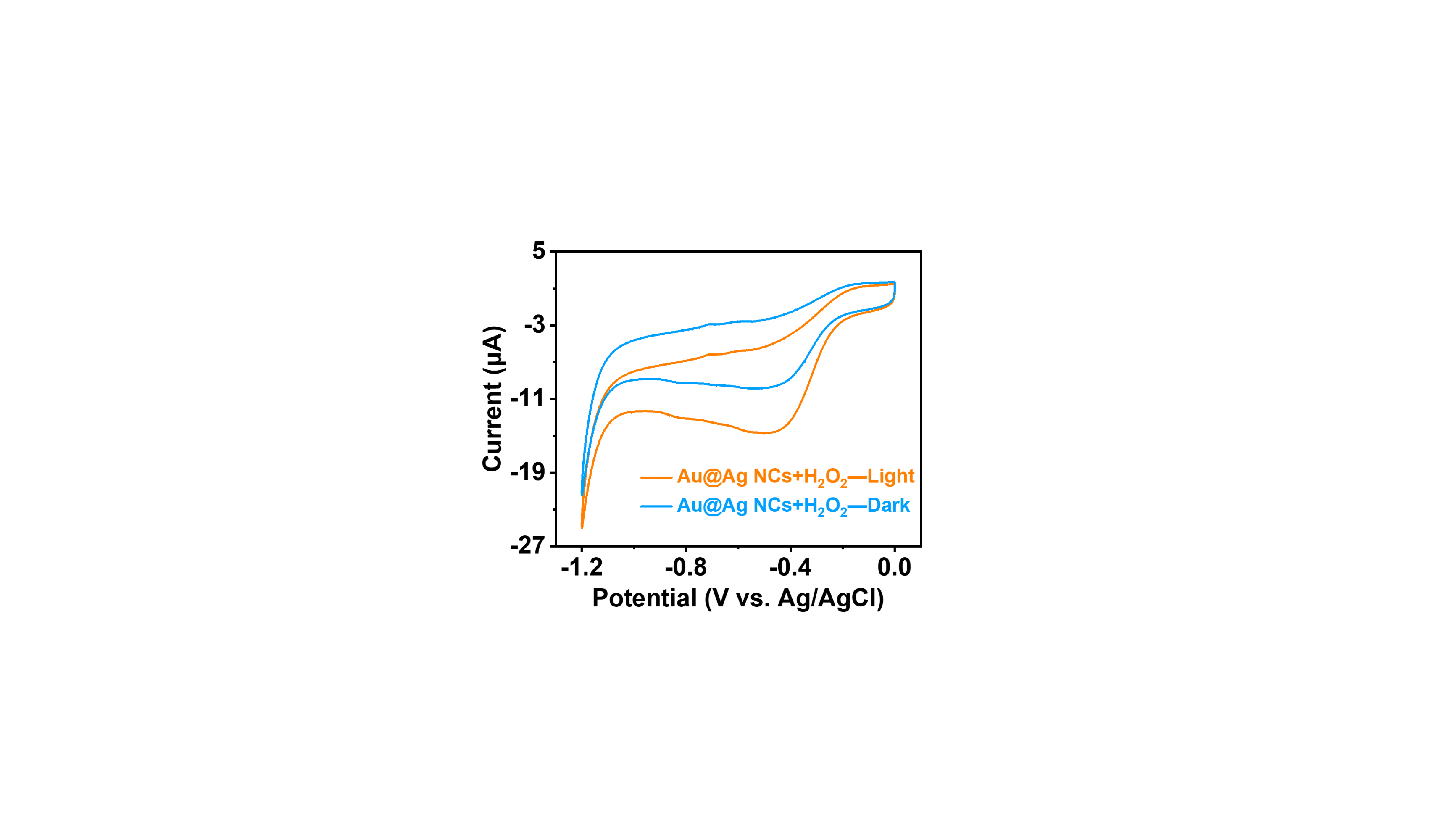


**Figure S20.** The CVs of AuAgNCs in 10 μmol L^-1^ H_2_O_2_ in dark or under light illumination with a wavelength of 450 nm.


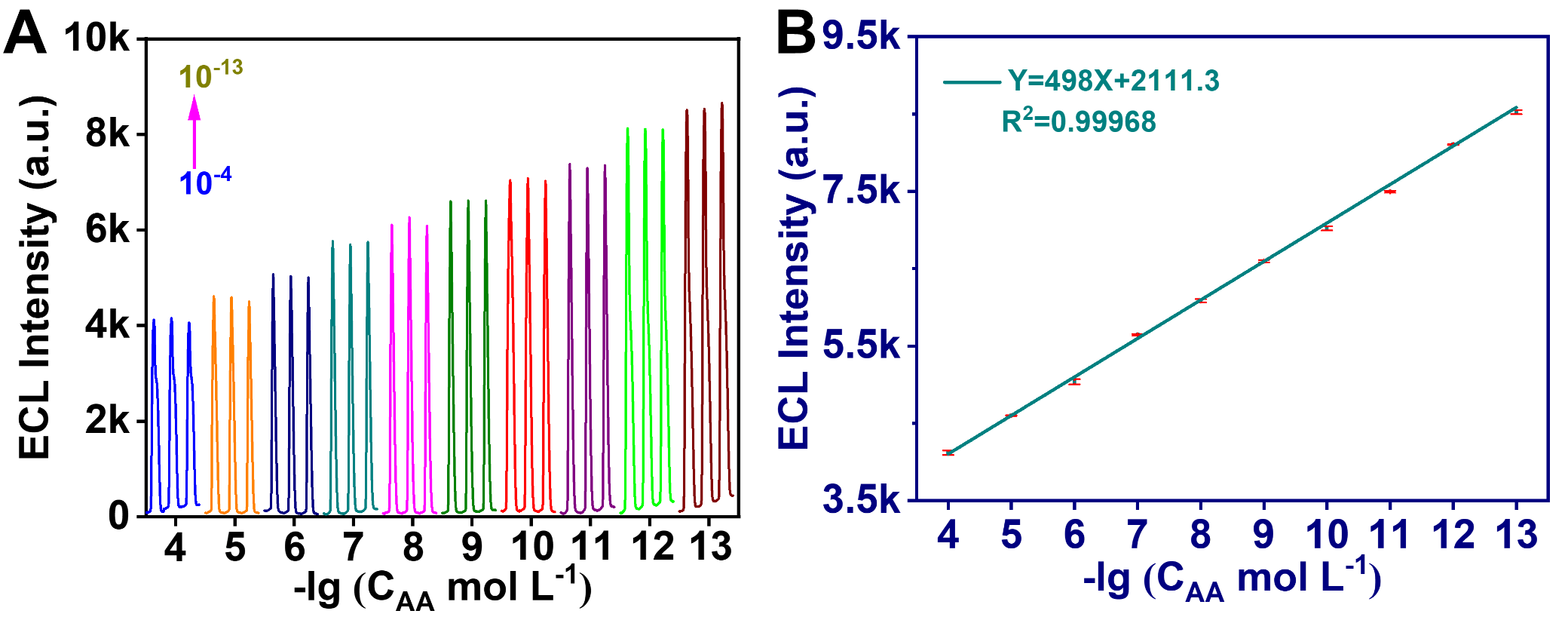


**Figure S21.** (A) ECL performances of CoNi-MOF@PCN-224/Fe with different concentrations of quencher (AA), and (B) the sensitivity of the ECL biosensor by using AA as cocatalyst quencher instead of AgAuNCs.

**Table S1.** Comparison of nanozyme activity with those previously reported.

| **No.** | **Nanozyme** | **K_m_(mM)** | **ν_max_(μM min^-1^)** | **Ref.** |
| --- | --- | --- | --- | --- |
| 1 | NCNzymes  PNCNzymes | 0.1412  0.07915 | 6.66  13.692 | Nano Today 2020, 35, 100935 |
| 2 | HccFn(Co_3_O_4_)  HccFn(Fe_3_O_4_) | 840  1120 | 10.98  3.06 | ACS Appl. Mater. Interfaces 2019, 11, 9747 |
| 3 | N-CDs  Fe-CDs | 0.156  0.09 | 4.29  10.30 | Nano Today 2022, 45, 101530 |
| 4 | PMCS | 0.224 | 6.396 | Angew. Chem., Int. Ed. 2019, 58, 4911 |
| 5 | N-nanozyme  Pero-nanozysome | 0.130  0.18 | 2.626  17.988 | Adv. Funct. Mater. 2021, 31, 2007130 |
| 6 | Ag-TiO_2_ SAN  TiO_2_  Ag | 0.032  0.223  0.068 | 52.02  8.34  29.52 | Nano Today 2021, 40, 101243 |
| 7 | CoNi-MOF@PCN-224/Fe | 1.23 | 15.96 | This work |

**Table S2.** Comparison of ECL detection performance with previously reported studies.

| **No.** | **Materials** | **LOD (pg mL^-1^)** | **Linear Range** | **Ref.** |
| --- | --- | --- | --- | --- |
| 1 | BPE array chip | 93 | 0.1-1000 ng mL^-1^ | Anal. Chem. 2022, 94, 3005 |
| 2 | Pt-tipped Au NRs-GO_x_ | 0.72 | 1.0 pg mL^-1^-10 ng mL^-1^ | Anal. Chem. 2019, 91, 12553 |
| 3 | Fc@MgAl-LDH | 0.034 | 0.05 pg mL^−1^-50 ng mL^−1^ | Biosens. Bioelectron. 2021, 190, 113437 |
| 4 | Au nanoclusters/Au-MnO_2_ | 1 | 10 fg mL^−1^ - 100 ng mL^−1^ | Sens. Actuators, B 2022, 369, 132397 |
| 5 | AuNP/GQDs-PEI-GO | 0.44 | 0.001 ng mL^−1^ - 100 ng mL^−1^ | Sens. Actuators, B 2020, 303, 127246 |
| 6 | InP/ZnS_MPA-MSA_ | 0.3 | 5 pg mL^−1^ -  1 ng mL^−1^ | Anal. Chem. 2022, 94, 1350 |
| 7 | ox-GCMs | 270 | 5-1000  Ng mL^-1^ | ACS Appl. Bio Mater. 2020, 3, 6358 |
| 8 | CoNi-MOF@PCN-224/Fe | 0.13 | 10^-4^-10^-13^g mL^-1^ | This work |

**Table S3.** Detection results in normal human serum spiked with different concentration of PSA.

| Sample number | Added (ng mL^−1^) | Detected (ng mL^−1^) | Recovery (%) |
| --- | --- | --- | --- |
| 1 | 0.100 | 0.10, SD=4.91 | 104.00 |
| 2 | 2.000 | 1.93, SD=3.72 | 96.50 |
| 3 | 10.000 | 10.51, SD=2.96 | 105.10 |

**Equ. S1:**

The mechanism of ·O_2_^-^ generation from H_2_O_2_ by the nanozyme could be descripted as below:^[3]^

Fe^3+^ + e^–^ → Fe^2+^
Fe^2+^ + H_2_O_2_ → Fe^3+^ + •OH + OH^–^
Fe^3+^ + H_2_O_2_ → Fe^2+^ + •O_2_H + H^+^

**References**

[1] a)G. Kresse, J. Furthmüller, *Phys. Rev. B* **1996**, 54, 11169; b)J. P. Perdew, K. Burke, M. Ernzerhof, *Phys. Rev. Lett.* **1996**, 77, 3865.

[2] P. E. Blöchl, *Phys. Rev. B* **1994**, 50, 17953.

[3] L. Shi, L. Q. Yang, H. B. Zhang, K. Chang, G. X. Zhao, T. Kako, J. H. Ye, *Appl. Catal. B* **2018**, 224, 60.
